# Supplementary material for: Identification of RING E3 pseudoligases in the TRIM protein family
Source: Nat Commun. 2025 Apr 11;16:3456. doi: 10.1038/s41467-025-58807-1 (PMC11992055; doi:10.1038/s41467-025-58807-1)
Supplement: Supplementary file 1 — Supplementary Information [file 41467_2025_58807_MOESM1_ESM.pdf]

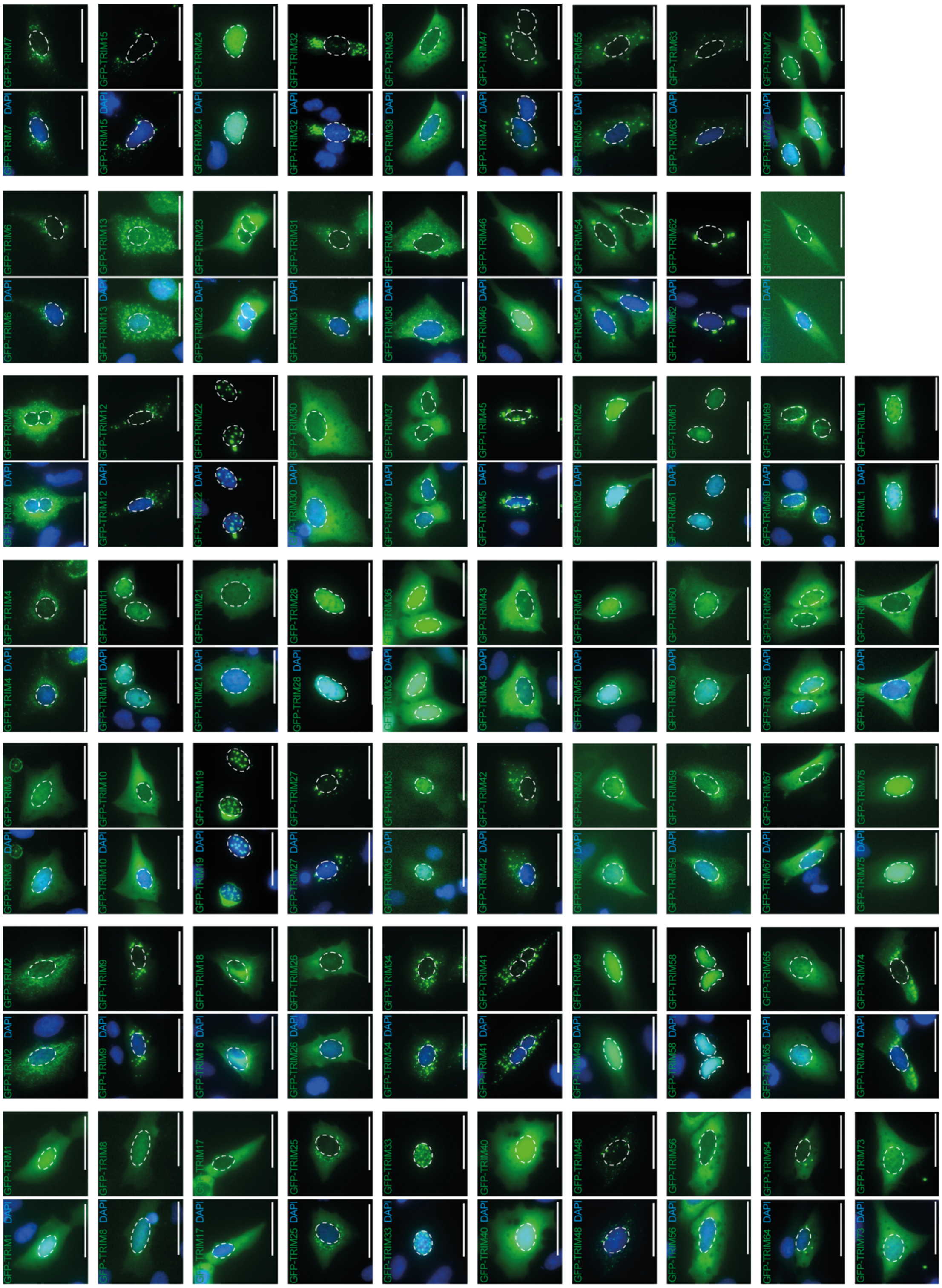

Supplementary Figure 1 – Localisation of RING-containing TRIM proteins

Representative images U2OS cells transiently over-expressing GFP-tagged RING-containing TRIM proteins 1 through to L1 (green), which were fixed and stained with DAPI (blue). Dashed lines denote the nucleus and the scale bars represent 50 µm. Images represent n=3.

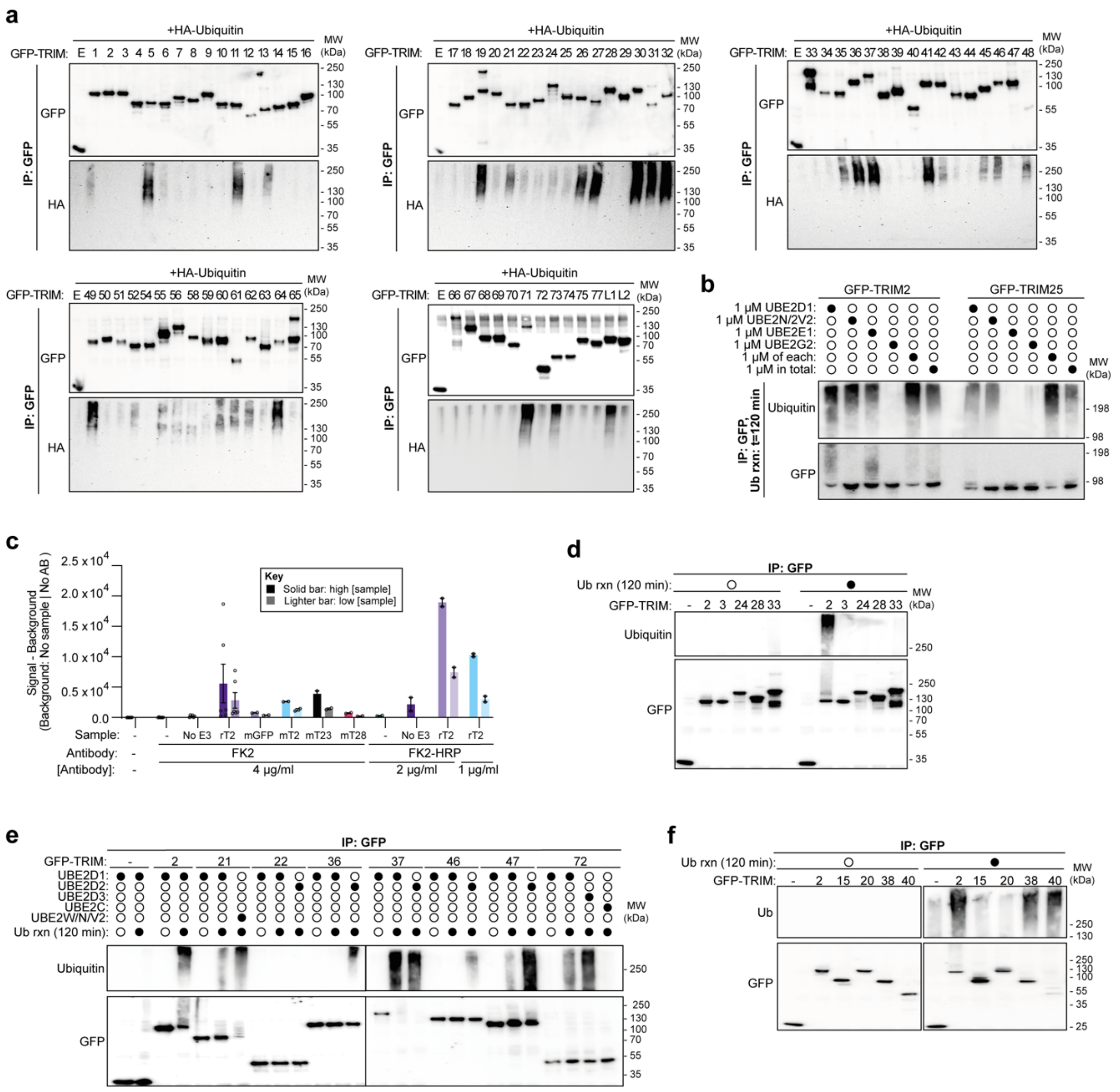

Supplementary Figure 2 – Data supporting screening TRIM family for in-cell and in vitro auto-ubiquitination activities

a, Relating to **Fig. 1f**, representative western blots using the indicated antibodies to detect in-cell TRIM ubiquitination using immunoprecipitated GFP-TRIMs transiently co-overexpressed with HA-Ubiquitin in HEK293T cells, treated with 10 μM PR619 and 10 μM MG132 4 h before lysis (n=3). **b**, Western blots using the indicated antibodies, demonstrating the efficacy of mixing E2 enzymes for in vitro auto-ubiquitination assays with GFP-tagged TRIM2 and TRIM25 isolated from HEK293T cells (n=2). **c,** Graph representing the validation of an ELISA assay specific to conjugated ubiquitin for assessment of in vitro auto-ubiquitination, using reaction mix without E3 or GFP only (negative controls), recombinant

TRIM2 RING domain (rT2: positive control) and full-length mammalian cell-derived TRIMs (mT2, mT23, or mT28) at high or low dilutions in the assay. FK2 antibody followed by anti-mouse-HRP was used to trial an indirect ELISA, whilst FK2-HRP was able to demonstrate the enhanced sensitivity of a direct ELISA (n=2). Error bars: mean ± SEM. **d**, Western blot using the indicated antibodies against immunoprecipitated GFP-tagged TRIM proteins from HEK293T cells and then used in an in vitro ubiquitination assay for 0 or 120 min with a mix of E2 enzymes as in **Fig. 1f** (n=3). **e**, Western blot using the indicated antibodies against immunoprecipitated GFP-tagged TRIM proteins purified from HEK293T cells, which were then used in an in vitro ubiquitination assay for 0 or 120 min with different E2 enzymes (UBE2C, UBE2D1, UBE2D2, UBE2D3, or a mix of UBE2N/UBE2V2/UBE2W) (n=3). **f**, Western blot using the indicated antibodies against immunoprecipitated GFP-tagged TRIM proteins purified from HEK293T cells, which were then used in an in vitro ubiquitination assay for 0 or 120 min with the mix of E2s as specified in **Fig. 1f** (n=3). Source data are provided as a Source Data file.

TRIM class	Class members	RING overview<br>AlphaFold2	Core RING<br>AlphaFold2	Notable predicted features
Class I	TRIM1<br>TRIM9<br>TRIM18<br>TRIM36<br>TRIM46<br>TRIM67	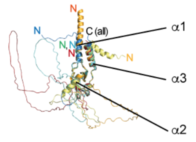	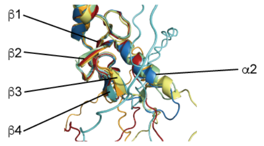	TRIM9, TRIM36, TRIM46, TRIM67<br>Long loop between α2 and β3
Class II	TRIM54<br>TRIM55<br>TRIM63	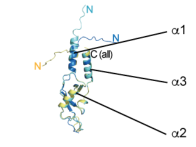	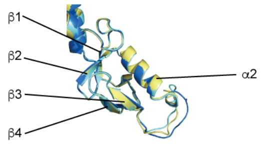	
Class III	TRIM42	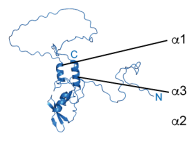	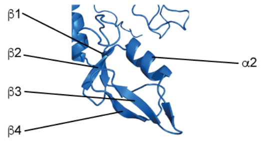	TRIM42<br>Long N-terminus preceding RING domain
Class IV	TRIM4 TRIM26 TRIM50<br>TRIM5* TRIM27 TRIM51<br>TRIM6 Trim30 TRIM58<br>TRIM7 TRIM34 TRIM60<br>TRIM10 TRIM35 TRIM62<br>TRIM11 TRIM38 TRIM64<br>Trim12 TRIM39 TRIM65<br>TRIM15 TRIM41 TRIM68<br>TRIM17 TRIM43 TRIM69*<br>TRIM21* TRIM47 TRIM72<br>TRIM22 TRIM48 TRIM75<br>TRIM25* TRIM49 TRIM77<br>TRIML1	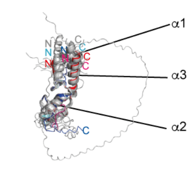	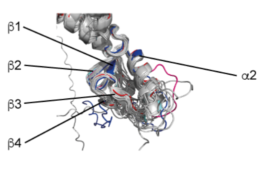	TRIM6<br>Unfolded α2<br>TRIM15<br>Unfolded α1, 2, 3 and β3, 4<br>TRIM51<br>Unfolded β3, 4<br>TRIM41<br>Long loop between α2 and β3<br>See Fig. 2
Class V	TRIM8<br>TRIM19*<br>TRIM31<br>TRIM40<br>TRIM52<br>TRIM56*<br>TRIM61<br>TRIM73<br>TRIM74	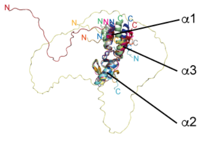	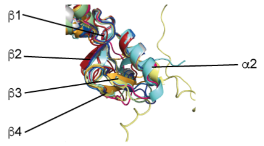	TRIM8, TRIM19, TRIM40<br>Unfolded β3, 4<br>TRIM52<br>Long loop between α2 and β3<br>TRIM19<br>Long N-terminus preceding RING domain
Class VI	TRIM24<br>TRIM28*<br>TRIM33	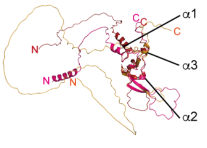	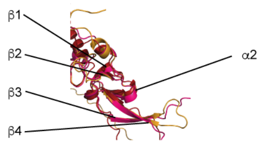	TRIM24, TRIM28<br>Largely unfolded α1, 2, 3<br>TRIM33<br>Unfolded α1, largely unfolded α2, 3<br>All<br>Long N-terminus preceding RING domain
Class VII	TRIM2*<br>TRIM3<br>TRIM32*<br>TRIM71	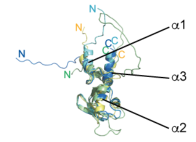	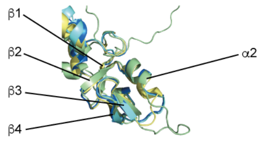	TRIM3<br>Unfolded α1
Class VIII	TRIM37*	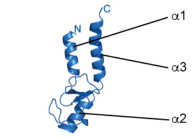	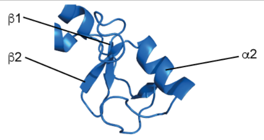	TRIM37<br>Unfolded β3, 4
Class IX	TRIM23*	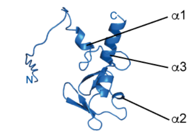	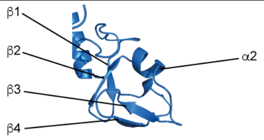	
Class X	TRIM45	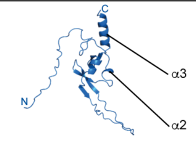	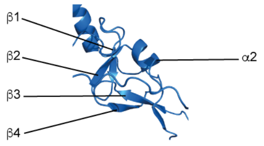	TRIM45<br>Unfolded α1, long loop between α2 and β3
Class XI	TRIM13<br>TRIM59	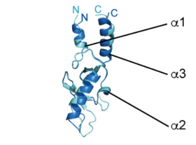	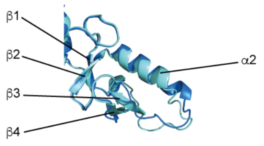	TRIM59<br>Unfolded β3, 4

Supplementary Figure 3 – AlphaFold2 *in silico* analyses of TRIM family proteins highlights notable structural differences, including in TRIMs identified as inactive

Table of AlphaFold2 structural predictions of TRIM RING domains (N-terminal Met to end of α3 helix, or with a sequence of corresponding length where no α3 helix is predicted), aligned and arranged according to class designation, with descriptions of notably divergent structural features given for some TRIMs. * indicates TRIMs with a RING domain structure available in the PDB (TRIM2: 7ZJ3; TRIM5: 4TKP; TRIM19: 5YUF; TRIM21: 5OLM, 6FGA, 7BBD, 6S53; TRIM23: 5CZV; TRIM25: 5EYA, 5FER; TRIM28: 6QAJ, 6I9H; TRIM32: 5FEY; TRIM37: 3LRQ; TRIM56: 5JW7; TRIM69: 6YXE).

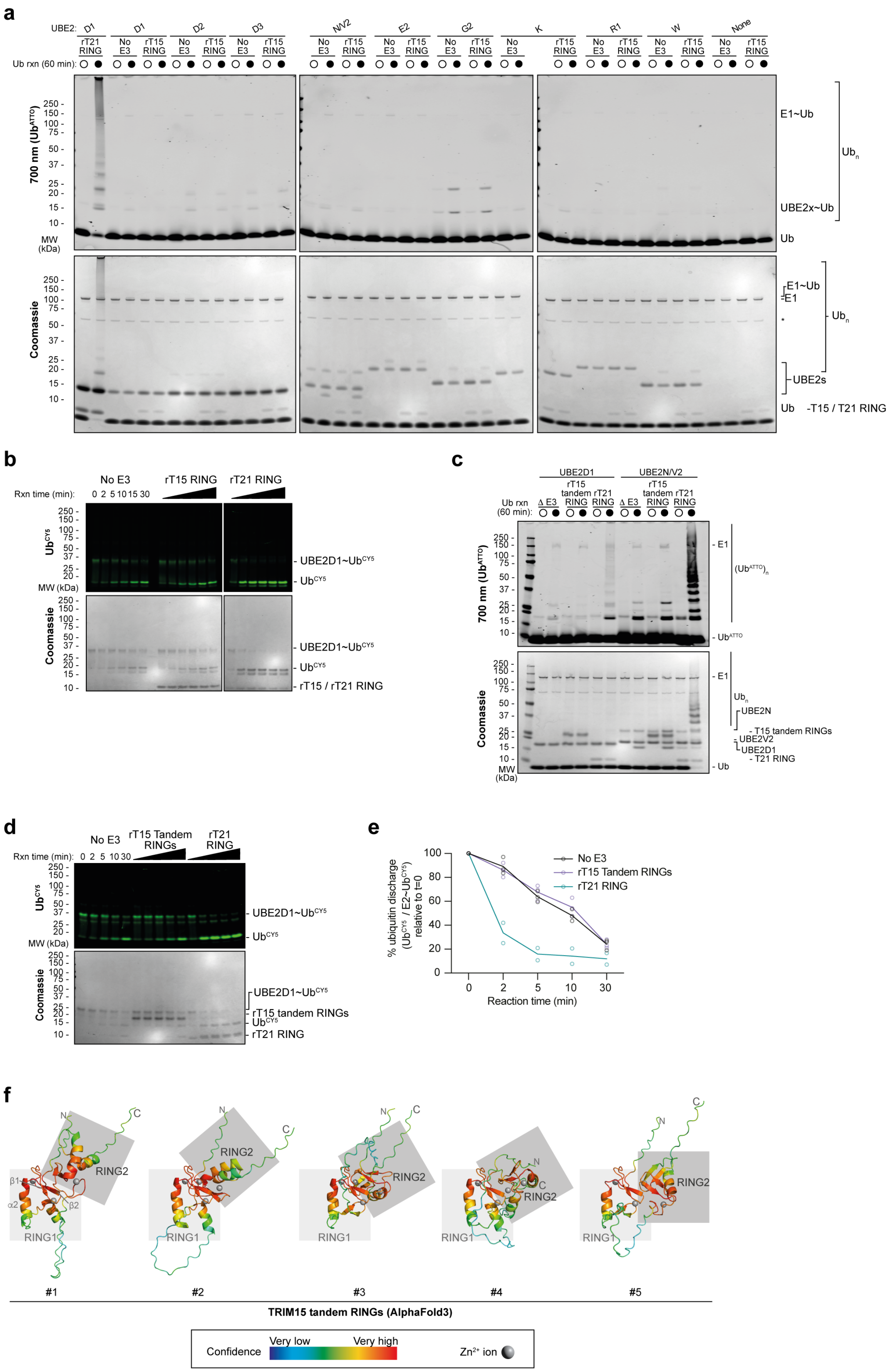
a
UBE2: D1 D1 D2 D3 N/V2 E2 G2 K R1 W None
rT21 RING
No E3
rT15 RING
Ub rxn (60 min):
700 nm (Ub^ATTO)
MW (kDa)
250 - 150 - 100 - 75 - 50 - 37 - 25 - 20 - 15 - 10 -
E1~Ub
Ubn
UBE2x~Ub
Ub
Coomassie
E1~Ub
E1
*
UBE2s
Ub
-T15 / T21 RING
b
No E3
rT15 RING
rT21 RING
Rxn time (min): 0 2 5 10 15 30
Ub^CY5
MW (kDa)
- UBE2D1~Ub^CY5
- Ub^CY5
Coomassie
- rT15 / rT21 RING
c
UBE2D1
UBE2N/V2
Δ E3
rT15 tandem RING
rT21 RING
Ub rxn (60 min):
700 nm (Ub^ATTO)
- E1
(Ub^ATTO)n
- Ub^ATTO
Coomassie
Ubn
UBE2N
- T15 tandem RINGs
UBE2V2
UBE2D1
- T21 RING
- Ub
MW (kDa)
d
No E3
rT15 Tandem RINGs
rT21 RING
Rxn time (min): 0 2 5 10 30
Ub^CY5
MW (kDa)
- UBE2D1~Ub^CY5
- Ub^CY5
Coomassie
UBE2D1~Ub^CY5
rT15 tandem RINGs
Ub^CY5
rT21 RING
e
% ubiquitin discharge (Ub^CY5 / E2~Ub^CY5) relative to t=0
No E3
rT15 Tandem RINGs
rT21 RING
Reaction time (min)
0 2 5 10 30
0 20 40 60 80 100
f
N C
RING1 RING2
β1 α2 β2
#1 #2 #3 #4 #5
TRIM15 tandem RINGs (AlphaFold3)
Confidence Very low Very high
Zn²⁺ ion

Supplementary Figure 4 – Data supporting TRIM15 has a monomeric RING domain that does not demonstrate ubiquitin ligase activity in isolation

a, Fluorescent and Coomassie gel scans of auto-ubiquitination reaction carried out at 30 °C 60 min using 1 μM of each of the indicated E2 enzymes (UBE2R1, D1, D2, D3, N/V2, W, G2, K, or E2) with 1 μM UBA1, 50 μM ubiquitin, 1 μM UbATTO, and 3 mM ATP, using no E3 or 4 μM of recombinant TRIM23 RBCC or TRIM15 RING (n=2). **b**, Fluorescent and Coomassie gel scans showing the discharge of Ub^{CY5} from pre-charged UBE2D1 onto free lysine in solution, with 4 μM recombinant TRIM21 RING domain or recombinant TRIM15 RING domain, or with no E3 (see **Fig. 3b**, n=3). **c,** Fluorescent and Coomassie gel scans of auto-ubiquitination reaction carried out at 30 °C 60 min using 1 μM of UBE2D1 or UBE2N/V2 with 1 μM UBA1, 50 μM ubiquitin, 1 μM UbATTO, and 3 mM ATP, using no E3 or 4 μM of recombinant TRIM21 RING or TRIM15 tandem RING domains (n=3). **d**, Fluorescent and Coomassie gel scans showing the discharge of Ub^{CY5} from pre-charged UBE2D1 onto free lysine in solution, with 4 μM recombinant TRIM21 RING domain or recombinant TRIM15 tandem RING domains, or with no E3 (n=3). **e**, Quantification of n=3 repeats of the experiment in part **d**. **f**, Top 5 AlphaFold3 models of TRIM15 tandem RING construct (with 4 x Zn^{2+}, grey), aligned according to α2 of the first RING domain, with the second RING domain demonstrating 5 distinct conformations, as highlighted by the grey boxes (light grey: RING1 orientation, dark grey: RING2 orientation). Source data are provided as a Source Data file.

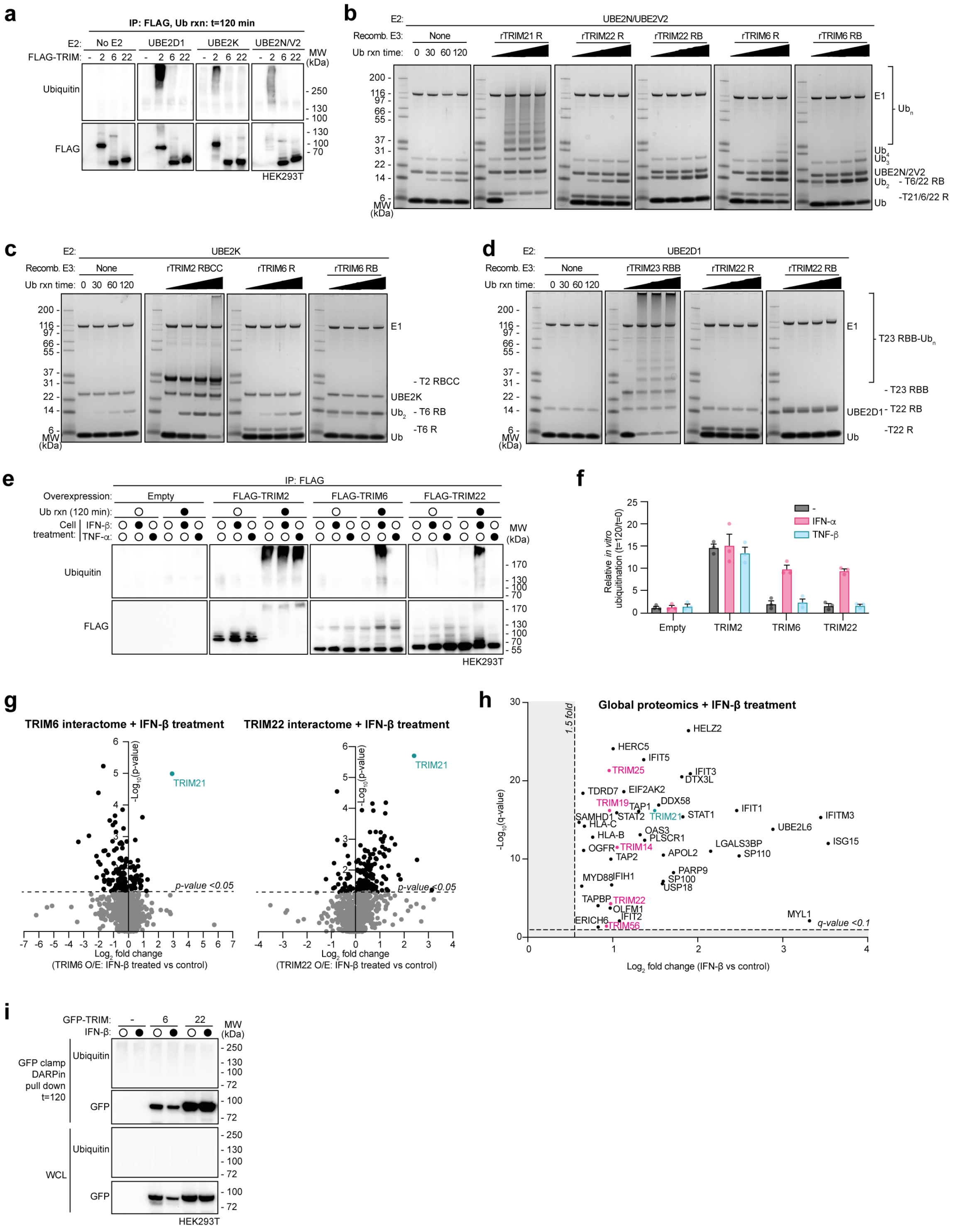

Supplementary Figure 5 – Analysis of TRIM6 and TRIM22 activity in response to interferon signalling

a, Western blots using the indicated antibodies against immunoprecipitated FLAG-tagged TRIM proteins purified from HEK293T cells, which were then used in an in vitro ubiquitination assay for 120 min with 1 μM of specified E2 enzymes (or no E2 control) with 1 μM UBA1, 50

μM ubiquitin, and 3 mM ATP (n=3). **b-d**, Coomassie gels of in vitro auto-ubiquitination assays for the indicated times as described in part a, excepting that 2 μM UBE2N/UBE2V2 (**b**), UBE2K (**c**), or UBE2D1 (**d**) was used, with recombinant TRIM21 RING (R), TRIM2 RBCC, or TRIM23 RBB as positive controls alongside either TRIM6 or TRIM22 R or RB (n=3). **e**, Western blots of auto-ubiquitination reaction carried out at 30 °C 120 min using 1 μM UBE2D1, 1 μM UBA1, 50 μM ubiquitin, and 3 mM ATP, using FLAG pull downs from either untransfected, FLAG-TRIM2, 6, or 22 transfected HEK293T cells that were either untreated or treated 18 h with 5,000 units/ml IFN-β or 20 ng/ml TNF-α (n=3). **f**, Quantification of n=3 independent experiments as described in part e, with individual values plotted as circles, error bars represent mean ± SEM. **g**, Volcano plots indicating the IFN-β-responsive interactomes of FLAG-TRIM6 (left) and FLAG-TRIM22 (right) (n=3), as determined by timsTOF mass spectrometry of anti-FLAG immunoprecipitated complexes from transfected HEK293T cells (5,000 units/ml IFN-β 18 h vs. untreated), with TRIM21 identified as a hit in both cases (teal). **h**, Volcano plot of global proteomic changes in HEK293T cells following treatment with 5,000 units/ml IFN-β 18 h (n=3). **i**, Pull down using 'GFP clamp' DARPin pull down against GFP-tagged TRIM6 and TRIM22 expressed in HEK293T cells, with or without pre-treatment with 5,000 units/ml IFN-β 18 h, before use in a ubiquitination reaction as described in part **e**. Source data are provided as a Source Data file.

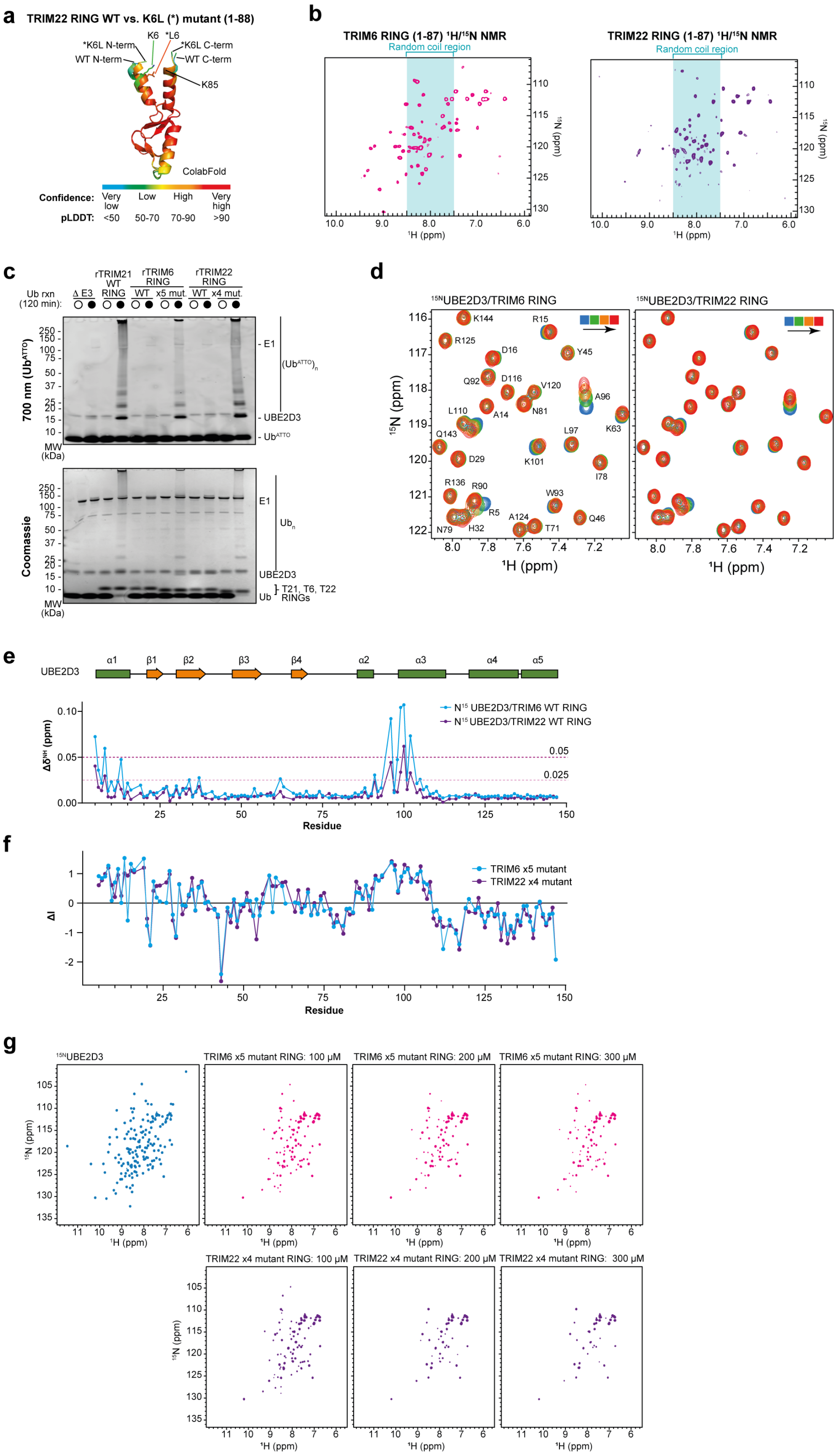

Supplementary Figure 6 – Data supporting key interfaces with E2~ubiquitin are mutated in TRIM6 and TRIM22, leading to lack of ubiquitin ligase activity in vitro

a, Alignments of ColabFold70 predictions of wild-type (WT) and K6L mutant TRIM22 (residues 1-88), demonstrating increased confidence in well-folded and closer a1 and a3 helices. **b**, ^{1}H-^{15}N HSQC spectra of TRIM6 (left, pink) and TRIM22 (right, purple) RING domains, with the spectral regions corresponding to resonances characteristic of residues in random coil conformation indicated in teal. **c**, 700 nm fluorescent imaging and Coomassie stains of auto-ubiquitination reactions carried out at 30 °C 120 min using 2 µM UBE2D3 with 1 µM UBA1, 50 µM ubiquitin, 1 µM UbATTO and 3 mM ATP, with recombinantly purified RING wild-type (WT) or active mutants (TRIM6 P41A/N42V/G43I/R54S/Q60R 'x5 mutant'; TRIM22 K6L/K42V/Q60R/K85V 'x4 mutant') TRIM6 or TRIM22 (n=3). **d**, Details of the ^{1}H-^{15}N HSQC spectra of UBE2D3 titrated with the RING domain of TRIM6 and TRIM22 WT. Spectra from blue to red at different ligand concentrations (0, 100, 200, 300 µM) are plotted at the same contour level. **e**, Chemical shift perturbations ($\Delta\delta NH$) versus residue number in the NMR spectra of ^{15}N-labelled UBE2D3 in the presence of two molar equivalents (300 µM) of the RING domains of TRIM6 (blue) and TRIM22 (purple). Secondary structure of UBE2D3 is reported as a function of residue number. **f**, Residue-specific values of differential line broadening (DI) of a 150 µM sample of ^{15}Nlabelled UBE2D3 induced by x5 mutant TRIM6 (blue) and x4 mutant TRIM22 (purple) at 100 µM concentration of RING domains. Residues with positive values above DI were mapped on the surface of UBE2D3 (**Fig. 4h**). **g**, Side-by-side comparison of the ^{1}H-^{15}N HSQC spectra of ^{15}N-labelled UBE2D3 in the absence (blue) or presence of the RING domain of x5 mutant TRIM6 (pink) and x4 mutant TRIM22 (purple) at increasing concentrations. Source data is provided as a Source Data file. Source data are provided as a Source Data file.

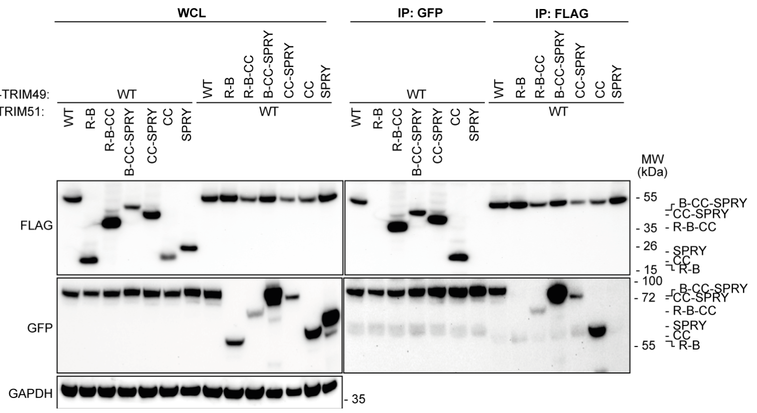

Supplementary Figure 7 – Data supporting TRIM49 and TRIM51 form an active:inactive pair that cross-regulate, with conflicting effects on autophagy

Western blot analysing the domains of FLAG-TRIM49 and GFP-TRIM51 that interact using co-immunoprecipitation of the indicated truncation mutant proteins from HEK293T cells (n=3, see **Fig. 5h**). Source data are provided as a Source Data file.

Supplementary Tables

Supplementary Table 1 – Comparative analysis of TRIM localisation identified in this study compared to that found previously in the literature, with any discrepancies highlighted in grey and the likely cause suggested

TRIM	Localisation		
	**Literature**	**This study**	**Cause of discrepancy** (listed respective to literature references)
1	Cytoskeleton[1-4]	Diffuse, nuclear	Different cell types (HeLa, H1299, Cos7, COS-1 cells)
2	Cytoskeleton[1,5]	Cytoskeleton	-
3	Diffuse[1,6]	Diffuse	-
4	Puncta[1,7], mitochondria[7]	Puncta	-
5	Puncta[1,8,9]	Puncta	-
6	Puncta[1,10]	Puncta	-
7	Diffuse[1,11], puncta[12], nuclear[1]	Puncta	-
8	Diffuse[13], puncta[1], nuclear[1,13]	Diffuse, nuclear	-
9	Puncta[1,14]	Puncta, aggregates	-
10	Diffuse[15], aggregates[1]	Diffuse	-
11	Diffuse[1], nuclear[1]	Diffuse, nuclear	-
12	Diffuse[1], puncta[1,16,17]	Puncta	-
13	Endoplasmic reticulum[1,18]	Puncta	Different cell type (HeLa) or endogenous staining
15	Puncta[19], cytoskeleton[20]	Puncta, aggregates	-
17	Diffuse[21]	Diffuse	-
18	Diffuse[14], cytoskeleton[1,22]	Cytoskeleton	-
19	Nuclear[1,23]	Nuclear	-
21	Diffuse[1], puncta[1,24]	Diffuse, puncta	-
22	Diffuse[1], puncta[1,25], nuclear[25,26]	Nuclear	-
23	Diffuse[27], puncta[1,27], nuclear[1]	Diffuse	-
24	Puncta[1], nuclear[28,29]	Nuclear	-
25	Diffuse[1], puncta[30], aggregates[1]	Puncta	-
26	Diffuse[1,31,32], puncta[33], nuclear[32]	Diffuse	-
27	Puncta[1,34], nuclear[34,35]	Puncta	-
28	Nuclear[1,36,37]	Nuclear	-
30	Diffuse[17], puncta[1,38], aggregates[17,38]	Diffuse	-
31	Diffuse[1], puncta[39-41]	Puncta	-
32	Puncta[1,14,42-45], nuclear[46]	Puncta, aggregates	-
33	Diffuse[47], nuclear[47-49]	Nuclear	-
34	Puncta[50]	Puncta	-
35	Diffuse[51]	Diffuse, nuclear	-
36	Cytoskeleton[52,53]	Diffuse, nuclear	Different cell type (HeLa)
37	Diffuse[54-57], puncta[55], aggregates[58], nuclear[56]	Diffuse	-
38	Puncta[59]	Puncta	-
39	Diffuse[60]	Diffuse	-
40	Diffuse[61], nuclear[61]	Diffuse, nuclear	-
41	Diffuse[62-64], nuclear[62]	Aggregates	Different cell type (SH-SY5Y, A549) with endogenous staining, or different cell type (RAW264.7)
42	Untested	Puncta	-
43	Puncta[65]	Diffuse	Different cell type (HFF) stimulated with HSV-1
45	Diffuse[66,67]	Diffuse, aggregates	-
46	Cytoskeleton[52,68,69]	Diffuse, nuclear	Different cell type (HeLa) or endogenous protein in hippocampal neurones
47	Diffuse[70]	Diffuse, aggregates	-
48	Untested	Puncta	-
49	Untested	Diffuse, nuclear	-
50	Diffuse[71,72], puncta[73-76]	Diffuse	-

51	Untested	Diffuse, nuclear	-
52	Nuclear[77]	Nuclear	-
54	Untested	Diffuse, aggregates	-
55	Diffuse[78-80], puncta[78]	Puncta	-
56	Diffuse[81,82]	Diffuse	-
58	Diffuse[83,84]	Nuclear	Different cell types (A549, HCC827, or HeLa) and tag (FLAG or mCherry)
59	Diffuse[85]	Diffuse	-
60	Aggregates[86]	Diffuse, nuclear	Different cell type (CHO-K1)
61	Untested	Nuclear	-
62	Diffuse[87], aggregates[88]	Diffuse, aggregates	-
63	Puncta[89-91], mitochondria[92]	Puncta	-
64	Untested	Diffuse, aggregates	-
65	Diffuse[93-95]	Diffuse, nuclear	-
67	Diffuse[96,97], puncta[96,97]	Diffuse	-
68	Diffuse[98]	Diffuse	-
69	Diffuse[99], aggregates[100,101]	Cytoskeleton	Different cell type (HeLa) and tags (FLAG or Myc)
71	Puncta[102,103]	Diffuse	Different cell types (KH2, embryocarcinoma, or HeLa cells) with endogenous staining
72	Diffuse[104,105], nuclear[104]	Diffuse	-
73	Untested	Diffuse	-
74	Untested	Diffuse, aggregates	-
75	Nuclear[106]	Nuclear	-
77	Untested	Diffuse	-
L1	Untested	Diffuse, nuclear	-

Supplementary Table 2 – Comparative analysis of TRIM ubiquitin ligase activity identified in this study compared to that found previously in the literature, with any discrepancies highlighted in grey and the likely cause suggested

TRIM	*in vitro* auto-ubiquitination activity			In cell auto-ubiquitination activity		
	**Literature**	**This study**	**Likely cause of discrepancy** (listed respective to literature references)	**Literature**	**This study**	**Likely cause of discrepancy** (listed respective to literature references)
1	Yes[14]	Yes	-	Yes[2,14]	Yes	-
2	Yes[107]	Yes	-	Yes[107]	No	Different cell type (HeLa) and IP (His-Ub IP)
3	Yes[108]	No	Lacks key RING dimerisation residues	Yes[6,109,110]	No	Lacks key RING dimerisation residues
4	Yes[111]	Yes	-	Yes[7]	No	Substrate ubiquitination rather than auto-ubiquitination
5	Yes[8]	Yes	-	Yes[8,9]	Yes	-
6	Yes[10]	No	Substrate ubiquitination rather than auto-ubiquitination	Yes[10,112]	Yes	-
7	Yes[11,113,114]	Yes	-	Yes[11,12,113,114]	No	Substrate ubiquitination rather than auto-ubiquitination
8	Yes[13]	Yes	-	Yes[13,115]	No	Substrate ubiquitination rather than auto-ubiquitination
9	Yes[14]	Yes	-	Yes[116]	No	Unknown
10	Untested	Yes	-	Yes[15,117,118]	No	Substrate ubiquitination rather than auto-ubiquitination
11	Yes[14]	Yes	-	Yes[14]	Yes	-
12	Untested	Yes	-	Yes[16]	No	Different isoform (Trim12c rather than Trim12a)
13	Yes[18]	Yes	-	Yes[18]	Yes	-
15	Yes[119]	No	Substrate ubiquitination rather than auto-ubiquitination	Yes[120]	No	Substrate ubiquitination rather than auto-ubiquitination
17	Yes[121]	Yes	-	Yes[21,121]	No	Different cell type (COS7)
18	Yes[122]	Yes	-	Yes[22]	No	Substrate ubiquitination rather than auto-ubiquitination
19	Untested	Yes	-	No[23,123]	Yes	Different cell type (HeLa)
21	Yes[124]	Yes	-	Yes[24,125]	Yes	-
22	Yes[126]	No	Different E2 (activity shown with Ube2D2)	Yes[26,126]	Yes	-
23	Yes[127,128]	Yes	-	Yes[27,128-130]	Yes	-
24	Yes[28] / No[131]	No	-	Yes[28,132,133]	Yes	-
25	Yes[30,134-136]	Yes	-	Yes[134,135,137]	Yes	-
26	Yes[138-140]	Yes	-	Yes[31,33,138-141]	Yes	-
27	Yes[14,35,142,143]	Yes	-	Yes[14,34,142-146]	Yes	-
28	Yes[147] / No[131]	No	-	Yes[148]	No	Ubiquitin E3 ligase activity under debate
30	Untested	Yes	-	Yes[149]	Yes	-
31	Yes[40,41,150-152]	Yes	-	Yes[39,40,150-155]	Yes	-
32	Yes[14,45,136,156-158]	Yes	-	Yes[14,45,46,156,159,160]	Yes	-
33	Yes[161] / No[131]	No	-	Yes[161,162]	No	Ubiquitin E3 ligase activity under debate
34	Yes[163]	Yes	-	Yes[163]	No	Substrate ubiquitination rather than auto-ubiquitination
35	Yes[164]	Yes	-	Yes[164-167]	Yes	-
36	Yes[53]	No	Different E2 (activity shown with Ube2D2)	Yes[53,168]	Yes	-
37	Yes[57,58,169]	No	Different E2 (activity shown with Ube2D2)	Yes[54,55,58,169,170]	Yes	-
38	Untested	No	-	Yes[171-174]	No	Unknown
39	Yes[175]	Yes	-	Yes[176-178]	No	Substrate ubiquitination rather than auto-ubiquitination
40	Yes[179]	No	Substrate ubiquitination rather than auto-ubiquitination	Yes[179-181]	No	Substrate ubiquitination rather than auto-ubiquitination
41	Yes[63,64]	Yes	-	Yes[62-64,182]	Yes	-
42	Untested	Yes	-	Untested	Yes	-

43	Untested	Yes	-	Yes[65]	No	Substrate ubiquitination rather than auto-ubiquitination
45	Yes[66,183]	Yes	-	Yes[67,184]	Yes	-
46	Yes[183]	No	Substrate ubiquitination rather than auto-ubiquitination	Yes[183,185-187]	Yes	-
47	Yes[188]	No	Different E2 (activity shown with Ube2D2)	Yes[70,188-191]	No	Substrate ubiquitination rather than auto-ubiquitination
48	Yes[192]	Yes	-	Yes[192]	Yes	-
49	No[193]	Yes	Mammalian expression untested (purified from bacteria)	Yes[193]	Yes	-
50	Yes[76]	Yes	-	Yes[71-75,194]	No	Substrate ubiquitination rather than auto-ubiquitination
51	Untested	No	-	Untested	No	-
52	Yes[195]	Yes	-	Yes[196-198]	Yes	-
54	Yes[199,200]	Yes	-	Yes[201,202]	No	Substrate ubiquitination rather than auto-ubiquitination
55	Yes[203]	Yes	-	Yes[204]	Yes	-
56	Yes[205,206]	Yes	-	Yes[81,205]	Yes	-
58	Yes[83]	Yes	-	Yes[83,84,207-210]	Yes	-
59	Yes[211]	Yes	-	Yes[211-214]	Yes	-
60	Untested	Yes	-	Untested	Yes	-
61	Untested	Yes	-	Untested	Yes	-
62	Yes[88]	Yes	-	Yes[88,215,216]	Yes	-
63	Yes[90]	Yes	-	Yes[89,90]	Yes	-
64	Untested	Yes	-	Yes[217]	Yes	-
65	Yes[93,218]	Yes	-	Yes[93-95,218-220]	Yes	-
67	Untested	Yes	-	Yes[96,97,221]	No	Substrate ubiquitination rather than auto-ubiquitination
68	Yes[222]	Yes	-	Yes[98]	No	Substrate ubiquitination rather than auto-ubiquitination
69	Yes[100,223]	Yes	-	Yes[100,101,224]	No	Unknown
71	Yes[103]	Yes	-	Yes[103,225,226]	Yes	-
72	Yes[227,228]	No	Different E2 (activity shown with Ube2D3 or 2C)	Yes[228]	No	Substrate ubiquitination rather than auto-ubiquitination
73	Untested	Yes	-	Untested	Yes	-
74	Untested	Yes	-	Untested	Yes	-
75	Untested	Yes	-	Untested	Yes	-
77	Untested	Yes	-	Untested	No	-
L1	Untested	Yes	-	Untested	Yes	-

Supplementary Table 3 – TRIM RING sequences and AlphaFold2 references

TRIM	RING sequence	AlphaFold2 PDB
TRIM1	MGESPASVVLNASGGLFSLKMETLESELTCPICLELFEDPLLLPCAHSLCFSCAHRILVSSCSSGESIEPITAFQCPTCRYVISLNHRGLDGLKRNVTLQNIIDRFQKASVSGP	AF-Q9UJV3-F1-model_v4
TRIM2	MASEGTNIPSPVVRQIDKQFLICSICLERYKNPKVLPCLHTFCERCLQNYIPAHSLTLSCPVCRQTSILPEKGVAALQNNFFITNLMDVLQRTP	AF-Q9C040-F1-model_v4
TRIM3	MAKREDSPGPEVQPMDKQFLVCSICLDRYQCPKVLPCLHTFCERCLQNYIPAQSLTLSCPVCRQTSILPEQGVSALQNNFFISSLMEAMQQAP	AF-O75382-F1-model_v4
TRIM4	MEAEDIQEELTCPICLDYFQDPVSIECGHNFCRGCLHRNWAPGGGPFPCPECRHPSAPAALRPNWALARLTEKTQRRRLGPVP	AF-Q9C037-F1-model_v4
TRIM5	MASGILVNVKEEVTCPICLELLTQPLSLDCGHSFCQACLTANHKKSMLDKGESSCPVCRISYQPENIRPNRHVANIVEKLREVKLSP	AF-Q9C035-F1-model_v4
TRIM6	MTSPVLVDIREEVTCPICLELLTEPLSIDCGHSFCQACITPNGRESVIGQEGERSCPVCQTSYQPGNLRPNRHLANIVRRLREVVLGP	AF-Q9C030-F1-model_v4
TRIM7	MAAVGPRTGPGTGAEALALAAELQGEATCSICLELFREPVSVECGHSFCRACIGRCWERPGAGSVGAATRAPPFPLPCPQCREPARPSQLRPNRQLAAVATLLRRFSLP	AF-Q9C029-F1-model_v4
TRIM8	MAENWKNCFEEELICPICLHVFVEPVQLPCKHNFCRGCIGEAWAKDSGLVRCPECNQAYNQKPGLEKNLKLTNIVEKFNALHVEKP	AF-Q9BZR9-F1-model_v4
TRIM9	MEEMEEELKCPVCGSFYREPIILPCSHNLCQACARNILVQTPESESPQSHRAAGSGVSDYDYLDLDKMSLYSEADSGYGSYGGFASAPTTPCQKSPNGVRVFPPAMPPPATHLSPALAPVPRNSCITCPQCHRSLILDDRGLRGFPKNRVLEGVIDRYQQSKA	AF-Q9C026-F1-model_v4
TRIM10	MASAASVTSLADEVNCPICQGTLREPVTIDCGHNFCRACLTRYCEIPGPDLEESPTCPLCKEPFRPGSFRPNWQLANVVENIERLQLVSTLGLGE	AF-Q9UDY6-F1-model_v4
TRIM11	MAAPDLSTNLQEEATCAICLDYFTDPVMTDCGHNFCRECIRRCWGQPEGPYACPECRELSPQRNLRPNRPLAKMAEMARRLHP	AF-Q96F44-F1-model_v4
Trim12	MASQFMKNLKEEVTCPVCLNLMVKPVSADCGHTFCQGCITLYFESIKCDKKVFICPVCRISYQFSNLRPNRNVANIVERLKMFKPSP	AF-Q99PQ1-F1-model_v4
TRIM13	MELLEEDLTCPICCSLFDDPRVLPCSHNFCKKCLEGILEGSVRNSLWRPAPFKCPTCRKETSATGINSLQVNYSLKGIVEKYNKIKISP	AF-O60858-F1-model_v4
TRIM15	MPATPSLKVVHELPACTLCAGPLEDAVTIPCGHTFCRLCLPALSQMGAQSSGKILLCPLCQEEEQAETPMAPVPLGPLGE	AF-Q9C019-F1-model_v4
TRIM17	MEAVELARKLQEEATCSICLDYFTDPVMTTCGHNFCRACIQLSWEKARGKKGRRKRKGSFPCPECREMSPQRNLLPNRLLTKVAEMAQQHP	AF-Q9Y577-F1-model_v4
TRIM18	METLESELTCPICLELFEDPLLLPCAHSLCFNCAHRILVSHCATNESVESITAFQCPTCRHVITLSQRGLDGLKRNVTLQNIIDRFQKASVSGP	AF-O15344-F1-model_v4
TRIM19	MEPAPARSPRPQQDPARPQEPTMPPPETPSEGRQPSPSPSPTERAPASEEEFQFLRCQQCQAEAKCPKLLPCLHTLCSGCLEASGMQCPICQAPWPLGADTPALDNVFFESLQRRLSVYRQIVDAQ	AF-P29590-F1-model_v4
TRIM21	MASAARLTMMWEEVTCPICLDPFVEPVSIECGHSFCQECISQVGKGGGSVCPVCRQRFLLKNLRPNRQLANMVNNLKEISQEARE	AF-P19474-F1-model_v4
TRIM22	MDFSVKVDIEKEVTCPICLELLTEPLSLDCGHSFCQACITAKIKESVIISRGESSCPVCQTRFQPGNLRPNRHLANIVERVKEVKMSP	AF-Q8IYM9-F1-model_v4
TRIM23	MATLVVNKLGAGVDSGRQGSRGTAVVKVLECGVCEDVFSLQGDKVPRLLLCGHTVCHDCLTRLPLHGRAIRCPFDRQVTDLGDSGVWGLKKNFALLELLERLQNGP	AF-P36406-F1-model_v4
TRIM24	MEVAVEKAVAAAAAASAAASGGPSAAPSGENEAESRQGPDSERGGEAARLNLLDTCAVCHQNIQSRAPKLLPCLHSFCQRCLPAPQRYLMLPAPMLGSAETPPPVPAPGSPVSGSSPFATQVGVIRCPVCSQECAERHIIDNFFVKDTTEVP	AF-O15164-F1-model_v4
TRIM25	MAELCPLAEELSCSICLEPFKEPVTTPCGHNFCGSCLNETWAVQGSPYLCPQCRAVYQARPQLHKNTVLCNVVEQFLQADLAREP	AF-Q14258-F1-model_v4
TRIM26	MATSAPLRSLEEEVTCSICLDYLRDPVTIDCGHVFCRSCTTDVRPISGSRPVCPLCKKPFKKENIRPVWQLASLVENIERLKVDKGRQP	AF-Q12899-F1-model_v4
TRIM27	MASGSVAECLQQETTCPVCLQYFAEPMMLDCGHNICCACLARCWGTAETNVSCPQCRETFPQRHMRPNRHLANVTQLVKQLRTERP	AF-P14373-F1-model_v4
TRIM28	MAASAAAASAAAASAASGSPGPGEGSAGGEKRSTAPSAAASASASAAASSPAGGGAEALELLEHCGVCRERLRPEREPRLLPCLHSACSACLGPAAPAAANSSGDGGAAGDGTVVDCPVCKQQCFSKDIVENYFMRDSGSKAATDA	AF-Q13263-F1-model_v4
Trim30	MASSVLEMIKEEVTCPICLELLKEPVSADCNHSFCRACITLNYESNRNTDGKGNCPVCRVPYPFGNLRPNLHVANIVERLKGFKSIP	AF-P15533-F1-model_v4
TRIM31	MASGQFVNKLQEEVICPICLDILQKPVTIDCGHNFCLKCITQIGETSCGFFKCPLCKTSVRKNAIRFNSLLRNLVEKIQALQASEVQSKRKE	AF-Q9BZY9-F1-model_v4
TRIM32	MAAAAASHLNLDALREVLECPICMESFTEEQLRPKLLHCGHTICRQCLEKLLASSINGVRCPFCSKITRITSLTQLTDNLTVLKIIDTAGLSEAVGLLMCRSCGRRLP	AF-Q13049-F1-model_v4
TRIM33	MAENKGGGEAESGGGGSGSAPVTAGAAGPAAQEAEPPLTAVLVEEEEEEGGRAGAEGGAAGPDDGGVAAASSGSAQAASSPAASVGTGVAGGAVSTPAPAPASAPAPGPSAGPPPGPPASLLDTCAVCQQSLQSRREAEPKLLPCLHSFCLRCLPEPERQLSVPIPGGSNGDIQQVGVIRCPVCRQECRQIDLVDNYFVKDTSEAP	AF-Q9UPN9-F1-model_v4
TRIM34	MASKILLNVQEEVTCPICLELLTEPLSLDCGHSLCRACITVSNKEAVTSMGGKSSCPVCGISYSFEHLQANQHLANIVERLKEVKLSP	AF-Q9BYJ4-F1-model_v4
TRIM35	MERSPDVSPGPSRSFKEELLCAVCYDPFRDAVTLRCGHNFCRGCVSRCWEVQVSPTCPVCKDRASPADLRTNHTLNNLVEKLLREEAEGARWTSYR	AF-Q9UPQ4-F1-model_v4
TRIM36	MSESGEMSEFGYIMELIAKGKVTIKNIERELICPACKELFTHPLILPCQHSICHKCVKELLLTLDDSFNDVGSDNSNQSSPRLRLPSPSMDKIDRINRPGWKRNSLTPRTTVFPCPGCEHDVDLGERGINGLFRNFTLETIVERYRQAARA	AF-Q9NQ86-F1-model_v4
TRIM37	MDEQSVESIAEVFRCFICMEKLRDARLCPHCSKLCCFSCIRRWLTEQRAQCPHCRAPLQLRELVNCRWAEEVTQQLDTLQLCSLTKHE	AF-O94972-F1-model_v4
TRIM38	MASTTSTKKMMEEATCSICLSLMTNPVSINCGHSYCHLCITDFFKNPSQKQLRQETFCCPQCRAPFHMDSLRPNKQLGSLIEALKETDQE	AF-O00635-F1-model_v4
TRIM39	MAETSLLEAGASAASTAAALENLQVEASCSVCLEYLKEPVIIECGHNFCKACITRWWEDLERDFPCPVCRKTSRYRSLRPNRQLGSMVEIAKQLQAVKRKIRDE	AF-Q9HCM9-F1-model_v4
TRIM40	MIPLQKDNQEEGVCPICQESLKEAVSTNCGHLFCRVCLTQHVEKASASGVFCCPLCRKPCSEEVLG	AF-Q6P9F5-F1-model_v4
TRIM41	MAAVAMTPNPVQTLQEEAVCAICLDYFTDPVSIGCGHNFCRVCVTQLWGGEDEEDRDELDREEEEEDGEEEEVEAVGAGAGWDTPMRDEDYEGDMEEEVEEEEEGVFWTSGMSRSSWDNMDYVWEEEDEEEDLDYYLGDMEEEDLRGEDEEDEEEVLEEVEEEDLDPVTPLPPPPAPRRCFTCPQCRKSFPRRSFRPNLQLANMVQVIRQMHP	AF-Q8WV44-F1-model_v4
TRIM42	METAMCVCCPCCTWQRCCPQLCSCLCCKFIFTSERNCTCFPCPYKDERNCQFCHCTCSESPNCHWCCCSWANDPNCKCCCTASSNLNCYYYESRCCRNTIITFHKGRLRSIHTSSKTALRTGSSDTQVDEVKSIPANSHLVNHLNCPMCSRLRLHSFMLPCNHSLCEKCLRQLQKHAEVTENFFILICPVCDRSHCMPYSNKMQLPENYLHGRLTKRYMQEHGYLKWRFDRSSGP	AF-Q8IWZ5-F1-model_v4

TRIM43	MDSDFSHAFQKELTCVICLNYLVDPVTICCGHSFCRPCLCLSWEEAQSPANCPACREPSPKMDFKTNILLKNLVTIARKASLWQFLSSE	AF-Q96BQ3-F1-model_v4
TRIM45	MSENRKPLLGFVSKLTSGTALGNSGKTHCPLCLGLFKAPRLLPCLHTVCTTCLEQLEPFSVVDIRGGDSDTSSEGSIFQELKPRSLQSQIGILCPVCDAQVDLPMGGVKALTIDHLAVNDVMLESLRGE	AF-Q9H8W5-F1-model_v4
TRIM46	MAEGEDMQTFTSIMDALVRISTSMKNMEKELLCPVCQEMYKQPLVLPCTHNVCQACAREVLGQQGYIGHGGDPSSEPTSPASTPSTRSPRLSRRTLPKPDRLDRLLKSGFGTYPGRKRGALHPQVIMFPCPACQGDVELGERGLAGLFRNLTLERVVERYRQSVSVG	AF-Q7Z4K8-F1-model_v4
TRIM47	MDGSGPFSCPICLEPLREPVTLPCGHNFCLACLGALWPHRGASGAGGPGGAARCPLCQEPFPDGLQLRKNHTLSELLQLRQGSGP	AF-Q96LD4-F1-model_v4
TRIM48	MSRRIIVGTLQRTQRNMNSGISQVFQRELTCPICMNYFIDPVTIDCGHSFCRPCFYLNWQDIPILTQCFECIKTIQQRNLKTNIRLKKMASLARKASLWLFLSSE	AF-Q8IWZ4-F1-model_v4
TRIM49	MNSGILQVFQGELICPLCMNYFIDPVTIDCGHSFCRPCFYLNWQDIPFLVQCSECTKSTEQINLKTNIHLKKMASLARKVSLWLFLSSE	AF-P0CI25-F1-model_v4
TRIM50	MAWQVSLPELEDRLQCPICLEVFKEPLMLQCGHSYCKGCLVSLSCHLDAELRCPVCRQAVDGSSSLPNVSLARVIEALRLP	AF-Q86XT4-F1-model_v4
TRIM51	MNSGILQVFQRALTCPICMNYFLDPVTIDCGHSFCRPCLYLNWQDTAVLAQCSECKKTTRQRNLNTDICLKNMAFIARKASLRQFLSSE	AF-Q9BSJ1-F1-model_v4
TRIM52	MAGYATTPSPMQTLQEEAVCAICLDYFKDPVSISCGHNFCRGCVTQLWSKEDEEDQNEEEDEWEEEEDEEAVGAMDGWDGSIREVLYRGNADEELFQDQDDDELWLGDSGITNWDNVDYMWDEEEEEEEEDQDYYLGGLRPDLRIDVYREEEILEAYDEDEDEELYPDIHPPPSLPLPGQFTCPQCRKSFTRRSFRPNLQLANMVQIIRQMCP	AF-Q96A61-F1-model_v4
TRIM54	MNFTVGFKPLLGDAHSMDNLEKQLICPICLEMFSKPVVILPCQHNLCRKCANDVFQASNPLWQSRGSTTVSSGGRFRCPSCRHEVVLDRHGVYGLQRNLLVENIIDIYKQESSRP	AF-Q9BYV2-F1-model_v4
TRIM55	MSASLNYKSFSKEQQTMDNLEKQLICPICLEMFTKPVVILPCQHNLCRKCASDIFQASNPYLPTRGGTTMASGGRFRCPSCRHEVVLDRHGVYGLQRNLLVENIIDIYKQESTRP	AF-Q9BYV6-F1-model_v4
TRIM56	MVSHGSSPSLLEALSSDFLACKICLEQLRAPKTLPCLHTYCQDCLAQLADGGRVRCPECRETVPVPPEGVASFKTNFFVNGLLDLVKARACGDLRAG	AF-Q9BRZ2-F1-model_v4
TRIM58	MAWAPPGERLREDARCPVCLDFLQEPVSVDCGHSFCLRCISEFCEKSDGAQGGVYACPQCRGPFRPSGFRPNRQLAGLVESVRRLGLG	AF-Q8NG06-F1-model_v4
TRIM59	MHNFEEELTCPICYSIFEDPRVLPCSHTFCRNCLENILQASGNFYIWRPLRIPLKCPNCRSITEIAPTGIESLPVNFALRAIIEKYQQEDHP	AF-Q8IWR1-F1-model_v4
TRIM60	MEFVTALVNLQEESSCPICLEYLKDPVTINCGHNFCRSCLSVSWKDLDDTFPCPVCRFCFPYKSFRRNPQLRNLTEIAKQLQIRRSKRKRQKE	AF-Q495X7-F1-model_v4
TRIM61	MEFVTALADLRAEASCPICLDYLKDPVTISCGHNFCLSCIIMSWKDLHDSFPCPFCHFCCPERKFISNPQLGSLTEIAKQLQIRSKKRKRQE	AF-Q5EBN2-F1-model_v4
TRIM62	MACSLKDELLCSICLSIYQDPVSLGCEHYFCRRCITEHWVRQEAQGARDCPECRRTFAEPALAPSLKLANIVERYSSFP	AF-Q9BVG3-F1-model_v4
TRIM63	MDYKSSLIQDGNPMENLEKQLICPICLEMFTKPVVILPCQHNLCRKCANDIFQAANPYWTSRGSSVSMSGGRFRCPTCRHEVIMDRHGVYGLQRNLLVENIIDIYKQECSSRP	AF-Q969Q1-F1-model_v4
TRIM64	MDSDDLQVFQNELICCICVNYFIDPVTIDCGHSFCRPCLCLCSEEGRAPMRCPSCRKISEKPNFNTNVVLKKLSSLARQTRP	AF-A6NGJ6-F1-model_v4
TRIM65	MAAQLLEEKLTCAICLGLYQDPVTLPCGHNFCGACIRDWWDRCGKACPECREPFPDGAELRRNVALSGVLEVVRAGP	AF-Q6PJ69-F1-model_v4
TRIM67	MEEELKCPVCGSLFREPIILPCSHNVCLPCARTIAVQTPDGEQHLPQPLLLSRGSGLQAGAAAAASLEHDAAAGPACGGAGGSAAGGLGGGAGGGGDHADKLSLYSETDSGYGSYTPSLKSPNGVRVLPMVPAPPGSSAAAARGAACSSLSSSSSSITCPQCHRSASLDHRGLRGFQRNRLLEAIVQRYQQGRGAVP	AF-Q6ZTA4-F1-model_v4
TRIM68	MDPTALVEAIVEEVACPICMTFLREPMSIDCGHSFCHSCLSGLWEIPGESQNWGYTCPLCRAPVQPRNLRPNWQLANVVEKVRLLRLHP	AF-Q6AZZ1-F1-model_v4
TRIM69	MEVSTNPSSNIDPGDYVEMNDSITHLPSKVVIQDITMELHCPLCNDWFRDPLMLSCGHNFCEACIQDFWRLQAKETFCPECKMLCQYNNCTFNPVLDKLVEKIKKLP	AF-Q86WT6-F1-model_v4
TRIM71	MASFPETDFQICLLCKEMCGSPAPLSSNSSASSSSSQTSTSSGGGGGGPGAAARRLHVLPCLHAFCRPCLEAHRLPAAGGGAAGEPLKLRCPVCDQKVVLAEAAGMDALPSSAFLLSNLLDAVVATADEP	AF-Q2Q1W2-F1-model_v4
TRIM72	MSAAPGLLHQELSCPLCLQLFDAPVTAECGHSFCRACLGRVAGEPAADGTVLCPCCQAPTRPQALSTNLQLARLVEGLAQVP	AF-Q6ZMU5-F1-model_v4
TRIM73	MAWQVSLLELEDRLQCPICLEVFKESLMLQCGHSYCKGCLVSLSYHLDTKVRCPMCWQVVDGSSSLPNVSLAWVIEALRLP	AF-Q86UV7-F1-model_v4
TRIM74	MAWQVSLLELEDWLQCPICLEVFKESLMLQCGHSYCKGCLVSLSYHLDTKVRCPMCWQVVDGSSSLPNVSLAWVIEALRLP	AF-Q86UV6-F1-model_v4
TRIM75	MAVAAALTGLQAEAKCSICLDYLSDPVTIECGHNFCRSCIQQSWLDLQELFPCPVCRHQCQEGHFRSNTQLGRMIEIAKLLQSTKSNKRKQE	AF-A6NK02-F1-model_v4
TRIM77	MASAITQCSTSELTCSICTDYLTDPVTICCGHRFCSPCLCLLWEDTLTPNCCPVCREISQQMYFKRIIFAEKQVIPTRESVP	AF-I1YAP6-F1-model_v4
TRIML1	MSTADLMENLREELTCFICLDYFSSPVTTECGHSFCLVCLLRSWEEHNTPLSCPECWRTLEGPHFQSNERLGRLASIARQLRSQVLQSEDEQGSYGRMP	AF-Q8N9V2-F1-model_v4

Supplementary Table 4 – Plasmid and antibody reagents used in this study

**Antibody Database**				
**Antibody**	**Use**	**Manufacturer**	**Catalogue Ref**	**Clone**
αGFP	Western, IP	Roche	11814460001	7.1 and 13.1
αUbiquitin (FK2: conjugated ubiquitin-specific)	Western	Sigma	ST1200	FK2
αUbiquitin-HRP (FK2: conjugated ubiquitin-specific)	ELISA	Generon	SMC-214D-HRP	FK2
αFLAG-HRP	Western	Merck	A8592	Clone M2
αFLAG	IF and IP	Merck	F1804	Clone M2
αUbiquitin (Ubi: pan-ubiquitin)	Western	Invitrogen	13-1600	Ubi-1
αTRIM6	Western, IF	Protein Tech	11953-1-AP	Polyclonal
αTRIM22	Western, IF	Atlas Antibodies	HPA003575	Polyclonal
αGAPDH	Western	Millipore	MAB374	Clone 6C5
αHA-HRP	Western	Merck	12013819001	Clone 3F10
αLC3B	Western, IF	Sigma	L7543	Polyclonal
αRabbit-HRP	Western	Cell Signaling	7074	Polyclonal
αMouse-HRP	Western	Cell Signaling	7076	Polyclonal
αMouse-HRP	Western	Dako	P0447	Polyclonal
αMouse-Alexa594	IF	ThermoFisher	A11032	Polyclonal
αRabbit-Alexa488	IF	ThermoFisher	A11008	Polyclonal
**Plasmid Database**				
**Plasmid**	**NCBI Protein Isoform Ref.**	**~Mol. Weight** (untagged kDa)	**Species**	**Derivation (and source)**
**Bacterial expression**				
pET28-His_6-UBA1	NP_003325.2	118	*Homo sapiens*	N/A (Rittinger Lab, Francis Crick Institute[229])
pET52-His_6-UBE2C	NP_008950.1	20	*Homo sapiens*	N/A (Rittinger Lab, Francis Crick Institute[229])
pGEX-6P1-GST-UBE2D1	NP_003329.1	17	*Homo sapiens*	N/A (Rittinger Lab, Francis Crick Institute[229])
pET49b-GST-UBE2D2	NP_862821.1	17	*Homo sapiens*	N/A (Rittinger Lab, Francis Crick Institute[131])
pET49b-His_6-UBE2D3	NP_003331.1	17	*Homo sapiens*	N/A (Rittinger Lab, Francis Crick Institute[136])
pET49b-His_6-UBE2E1	NP_003332.1	21	*Homo sapiens*	N/A (Rittinger Lab, Francis Crick Institute[131])
pET49b-GST-UBE2G2	NP_003334.2	19	*Homo sapiens*	N/A (Rittinger Lab, Francis Crick Institute[229])
pET49b-His_6-UBE2K	NP_005330.1	22	*Homo sapiens*	N/A (Rittinger Lab, Francis Crick Institute[131])
pNH-CTH-His_6-UBE2N	NP_003339.1	17	*Homo sapiens*	N/A (Rittinger Lab, Francis Crick Institute[229])
pGEX-6P1-GST-UBE2V2	NP_003341.1	16	*Homo sapiens*	N/A (Rittinger Lab, Francis Crick Institute[229])
pET22b-His_6-UBE2W	NP_001001481.3	17	*Homo sapiens*	N/A (Rittinger Lab, Francis Crick Institute[229])
pET49b-His_6-TRIM6 RING	NP_477514.1	10	*Homo sapiens*	N/A (Rittinger Lab, Francis Crick Institute[229])
pET49b-His_6-TRIM6 RING P41A/N42V/G43I/R54S/Q60R	N/A (mutant)	10	N/A (mutant)	This study (see Methods)
pET49b-His_6-TRIM6 RB	NP_477514.1	15	*Homo sapiens*	N/A (Rittinger Lab, Francis Crick Institute[229])
pET49b-His_6-TRIM6 BCC	NP_477514.1	12	*Homo sapiens*	N/A (Rittinger Lab, Francis Crick Institute[229])
pET49b-His_6-Trx-TRIM6 RING	NP_477514.1	10	*Homo sapiens*	N/A (Rittinger Lab, Francis Crick Institute[229])
pET49b-His_6-TRIM22 RING	NP_006065.2	10	*Homo sapiens*	N/A (Rittinger Lab, Francis Crick Institute[229])
pET49b-His_6-TRIM22 RING K6L/K42V/Q60R/K85V	N/A (mutant)	10	N/A (mutant)	This study (see Methods)
pET49b-His_6-TRIM22 RB	NP_006065.2	15	*Homo sapiens*	N/A (Rittinger Lab, Francis Crick Institute[229])
pET49b-His_6-TRIM22 BCC	NP_006065.2	13	*Homo sapiens*	N/A (Rittinger Lab, Francis Crick Institute[229])
pET49b-His_6-SUMO-TRIM22 RING	NP_477514.1	10	*Homo sapiens*	N/A (Rittinger Lab, Francis Crick Institute[229])
pET49b-His_6-TRIM21 RING	NP_003132.2	10	*Homo sapiens*	N/A (Rittinger Lab, Francis Crick Institute[229])

pET49b-His$_6$-TRIM15 tandem RINGs	NP_150232.2		*Homo sapiens*	This study (see Methods)
pET49b-His$_6$-TRIM23 RBB	NP_001647.1	23	*Homo sapiens*	N/A (Rittinger Lab, Francis Crick Institute[229])
pET49b-His$_6$-TRIM15 R	NP_150232.2		*Homo sapiens*	This study (see Methods)
pCMV-HA-ubiquitin	NP_001029102.1	9	*Homo sapiens*	N/A (Dundee PPU , #DU48359)
pcDNA3.1-FLAG-TRIM15	NP_150232.2	52	*Homo sapiens*	Sub-cloned from ptCMV-EGFP-TRIM15 (see below)
pcDNA3.1-FLAG-TRIM49	NP_065091.1	53	*Homo sapiens*	Sub-cloned from ptCMV EGFP-TRIM49 (see below)
pcDNA3.1-FLAG-TRIM51	NP_116070.2	53	*Homo sapiens*	Sub-cloned from ptCMV-GFP-TRIM49 (see below)
pcDNA3.1-FLAG-TRIM49 RB (1-128)	NP_065091.1	15	*Homo sapiens*	Sub-cloned from ptCMV-EGFP-TRIM49 (see below)
pcDNA3.1-FLAG-TRIM49 RBCC (1-259)	NP_065091.1	31	*Homo sapiens*	Sub-cloned from ptCMV-EGFP-TRIM49 (see below)
pcDNA3.1-FLAG-TRIM49 BCCSPRY (88-450)	NP_065091.1	43	*Homo sapiens*	Sub-cloned from ptCMV-EGFP-TRIM49 (see below)
pcDNA3.1-FLAG-TRIM 9 CC (129-259)	NP_065091.1	16	*Homo sapiens*	Sub-cloned from ptCMV-EGFP-TRIM49 (see below)
pcDNA3.1-FLAG-TRIM49 CCSPRY (129-450)	NP_065091.1	37	*Homo sapiens*	Sub-cloned from ptCMV-EGFP-TRIM49 (see below)
pcDNA3.1-FLAG-TRIM49 SPRY (260-450)	NP_065091.1	22	*Homo sapiens*	Sub-cloned from ptCMV-EGFP-TRIM49 (see below)
ptCMV-EGFP-TRIM51 RB (1-128)	NP_116070.2	15	*Homo sapiens*	Sub-cloned from ptCMV-EGFP-TRIM51 (see below)
ptCMV-EGFP-TRIM51 RBCC (1-259)	NP_116070.2	31	*Homo sapiens*	Sub-cloned from ptCMV-EGFP-TRIM51 (see below)
ptCMV-EGFP-TRIM5 BCCSPRY (88-450)	NP_116070.2	43	*Homo sapiens*	Sub-cloned from ptCMV-EGFP-TRIM51 (see below)
ptCMV-EGFP-TRIM51 CC (129-259)	NP_116070.2	16	*Homo sapiens*	Sub-cloned from ptCMV-EGFP-TRIM51 (see below)
ptCMV-EGFP-TRIM51 CCSPRY (129-450)	NP_116070.2	37	*Homo sapiens*	Sub-cloned from ptCMV-EGFP-TRIM51 (see below)
ptCMV-EGFP-TRIM51 SPRY (260-450)	NP_116070.2	22	*Homo sapiens*	Sub-cloned from ptCMV-EGFP-TRIM51 (see below)
pcDNA3.1-FLAG-TRIM49 β3-β4 TRIM51 swap	N/A (mutant)	53	*Homo sapiens*	gBlock (IDT) of TRIM51 β3-β4 inserted by Gibson Assembly into WT TRIM49 vector, opened by PCR primers flanking the relevant region
pcDNA3.1-FLAG-TRIM51 β3-β4 TRIM49 swap	N/A (mutant)	53	*Homo sapiens*	gBlock (IDT) of TRIM49 β3-β4 inserted by Gibson Assembly into WT TRIM51 vector, opened by PCR primers flanking the relevant region
ptCMV-EGFP-empty	N/A	N/A	N/A	N/A (T. Thurston Laboratory, Imperial College London[230])
ptCMV-EGFP-TRIM1	NP_036348.2	55	*Homo sapiens*	PCR from 293ET human cDNA, ptCMV-EGFP plasmid derived from pEGFP-N1 plasmid (Clontech) (T. Thurston Laboratory, Imperial College London[230])
ptCMV-EGFP-TRIM2	NP_001123539.1	82	*Homo sapiens*	PCR from 293ET human cDNA , ptCMV-EGFP plasmid derived from pEGFP-N1 plasmid (Clontech) (T. Thurston Laboratory, Imperial College London[230])
ptCMV-EGFP-TRIM3	NP_001234935.1	82	*Homo sapiens*	Subcloned from pcDNA3.1-FLAG-TRIM3 (Rittinger Lab, Francis Crick Institute[229]), ptCMV-EGFP plasmid derived from pEGFP-N1 plasmid (Clontech) (T. Thurston

				Laboratory, Imperial College London[230])
ptCMV-EGFP-TRIM4	NP_148977.2	57	*Homo sapiens*	PCR from mixed human cDNA, ptCMV-EGFP plasmid derived from pEGFP-N1 plasmid (Clontech) (T. Thurston Laboratory, Imperial College London[230])
ptCMV-EGFP-TRIM5	NP_149023.2	55	*Homo sapiens*	PCR from mixed human cDNA, ptCMV-EGFP plasmid derived from pEGFP-N1 plasmid (Clontech) (T. Thurston Laboratory, Imperial College London[230])
ptCMV-EGFP-TRIM6	NP_001003818.1	56	*Homo sapiens*	PCR from mixed human cDNA, ptCMV-EGFP plasmid derived from pEGFP-N1 plasmid (Clontech) (T. Thurston Laboratory, Imperial College London[230])
ptCMV-EGFP-TRIM7	NP_976038.1	57	*Homo sapiens*	PCR from mixed human cDNA, ptCMV-EGFP plasmid derived from pEGFP-N1 plasmid (Clontech) (T. Thurston Laboratory, Imperial College London[230])
ptCMV-EGFP-TRIM8	NP_112174.2	62	*Homo sapiens*	PCR from mixed human cDNA, ptCMV-EGFP plasmid derived from pEGFP-N1 plasmid (Clontech) (T. Thurston Laboratory, Imperial College London[230])
ptCMV-EGFP-TRIM9	NP_055978.4	80	*Homo sapiens*	PCR from 293ET human cDNA, ptCMV-EGFP plasmid derived from pEGFP-N1 plasmid (Clontech) (T. Thurston Laboratory, Imperial College London[230])
ptCMV-EGFP-TRIM10	NP_006769.2	55	*Homo sapiens*	Subcloned from M6P-EGFP vector generated by PCR of mixed human cDNA, ptCMV-EGFP plasmid derived from pEGFP-N1 plasmid (Clontech) (T. Thurston Laboratory, Imperial College London[230])
ptCMV-EGFP-TRIM11	NP_660215.1	53	*Homo sapiens*	Subcloned from M6P-EGFP vector generated by PCR of mixed human cDNA, ptCMV-EGFP plasmid derived from pEGFP-N1 plasmid (Clontech) (T. Thurston Laboratory, Imperial College London[230])
ptCMV-EGFP-TRIM12	NP_001355683.1	53	*Mus musculus*	PCR from iBMDM murine cDNA, ptCMV-EGFP plasmid derived from pEGFP-N1 plasmid (Clontech) (T. Thurston Laboratory, Imperial College London[230])
ptCMV-EGFP-TRIM13	NP_005789.2	47	*Homo sapiens*	PCR from THP1 human cDNA, ptCMV-EGFP plasmid derived from pEGFP-N1 plasmid (Clontech) (T. Thurston Laboratory, Imperial College London[230])

ptCMV-EGFP-TRIM14	NP_055603.2	50	*Homo sapiens*	PCR from mixed human cDNA, ptCMV-EGFP plasmid derived from pEGFP-N1 plasmid (Clontech) (T. Thurston Laboratory, Imperial College London[230])
ptCMV-EGFP-TRIM15	NP_150232.2	52	*Homo sapiens*	PCR from mixed human cDNA, ptCMV-EGFP plasmid derived from pEGFP-N1 plasmid (Clontech) (T. Thurston Laboratory, Imperial College London[230])
ptCMV-EGFP-TRIM16	NP_001335048.1	64	*Homo sapiens*	PCR from mixed human cDNA , ptCMV-EGFP plasmid derived from pEGFP-N1 plasmid (Clontech) (T. Thurston Laboratory, Imperial College London[230])
ptCMV-EGFP-TRIM17	NP_001020111.1	54	*Homo sapiens*	PCR from mixed human cDNA, ptCMV-EGFP plasmid derived from pEGFP-N1 plasmid (Clontech) (T. Thurston Laboratory, Imperial College London[230])
ptCMV-EGFP-TRIM18	NP_000372.1	72	*Homo sapiens*	PCR from Caco2 human cDNA, ptCMV-EGFP plasmid derived from pEGFP-N1 plasmid (Clontech) (T. Thurston Laboratory, Imperial College London[230])
ptCMV-EGFP-TRIM19	NP_150241.2	98	*Homo sapiens*	PCR from mixed human cDNA, ptCMV-EGFP plasmid derived from pEGFP-N1 plasmid (Clontech) (T. Thurston Laboratory, Imperial College London[230])
ptCMV-EGFP-TRIM20	NP_000234.1	86	*Homo sapiens*	PCR from mixed human cDNA, ptCMV-EGFP plasmid derived from pEGFP-N1 plasmid (Clontech) (T. Thurston Laboratory, Imperial College London[230])
ptCMV-EGFP-TRIM21	NP_003132.2	54	*Homo sapiens*	PCR from U937 human cDNA, ptCMV-EGFP plasmid derived from pEGFP-N1 plasmid (Clontech) (T. Thurston Laboratory, Imperial College London[230])
ptCMV-EGFP-TRIM22	NP_006065.2	57	*Homo sapiens*	Subcloned from M6P-EGFP vector generated by PCR of U937 human cDNA, ptCMV-EGFP plasmid derived from pEGFP-N1 plasmid (Clontech) (T. Thurston Laboratory, Imperial College London[230])
ptCMV-EGFP-TRIM23	NP_001647.1	64	*Homo sapiens*	Subcloned from plasmid gifted from Michaela Gack[27] (T. Thurston Laboratory, Imperial College London)
ptCMV-EGFP-TRIM24	NP_056989.2	117	*Homo sapiens*	PCR from plasmid synthesised by Open Biosystems, ptCMV-EGFP plasmid derived from pEGFP-N1 plasmid (Clontech) (T. Thurston

				Laboratory, Imperial College London[230])
ptCMV-EGFP-TRIM25	NP_005073.2	71	*Homo sapiens*	PCR from mixed human cDNA, ptCMV-EGFP plasmid derived from pEGFP-N1 plasmid (Clontech) (T. Thurston Laboratory, Imperial College London[230])
ptCMV-EGFP-TRIM26	NP_001229712.1	62	*Homo sapiens*	PCR from U937 human cDNA, ptCMV-EGFP plasmid derived from pEGFP-N1 plasmid (Clontech) (T. Thurston Laboratory, Imperial College London[230])
ptCMV-EGFP-TRIM27	NP_006501.1	58	*Homo sapiens*	PCR from 293ET human cDNA, ptCMV-EGFP plasmid derived from pEGFP-N1 plasmid (Clontech) (T. Thurston Laboratory, Imperial College London[230])
ptCMV-EGFP-TRIM28	NP_005753.1	100	*Homo sapiens*	PCR from mixed human cDNA, ptCMV-EGFP plasmid derived from pEGFP-N1 plasmid (Clontech) (T. Thurston Laboratory, Imperial College London[230])
ptCMV-EGFP-TRIM29	NP_036233.2	66	*Homo sapiens*	PCR from mixed human cDNA, ptCMV-EGFP plasmid derived from pEGFP-N1 plasmid (Clontech) (T. Thurston Laboratory, Imperial College London[230])
ptCMV-EGFP-TRIM30	NP_001344396.1	105	*Mus musculus*	Subcloned from M6P-EGFP vector generated by PCR of iBMDM murine cDNA, ptCMV-EGFP plasmid derived from pEGFP-N1 plasmid (Clontech) (T. Thurston Laboratory, Imperial College London[230])
ptCMV-EGFP-TRIM31	NP_008959.3	48	*Homo sapiens*	PCR from mixed human cDNA, ptCMV-EGFP plasmid derived from pEGFP-N1 plasmid (Clontech) (T. Thurston Laboratory, Imperial College London[230])
ptCMV-EGFP-TRIM32	NP_001093149.1	72	*Homo sapiens*	PCR from mixed human cDNA, ptCMV-EGFP plasmid derived from pEGFP-N1 plasmid (Clontech) (T. Thurston Laboratory, Imperial College London[230])
ptCMV-EGFP-TRIM33	NP_056990.3	150	*Homo sapiens*	PCR from mixed human cDNA, ptCMV-EGFP plasmid derived from pEGFP-N1 plasmid (Clontech) (T. Thurston Laboratory, Imperial College London[230])
ptCMV-EGFP-TRIM34	NP_001003827.1	36	*Homo sapiens*	PCR from mixed human cDNA, ptCMV-EGFP plasmid derived from pEGFP-N1 plasmid (Clontech) (T. Thurston Laboratory, Imperial College London[230])
ptCMV-EGFP-TRIM35	NP_741983.2	55	*Homo sapiens*	PCR from mixed human cDNA, ptCMV-EGFP

				plasmid derived from pEGFP-N1 plasmid (Clontech) (T. Thurston Laboratory, Imperial College London[230])
ptCMV-EGFP-TRIM36	NP_061170.2	83	*Homo sapiens*	PCR from mixed human cDNA, ptCMV-EGFP plasmid derived from pEGFP-N1 plasmid (Clontech) (T. Thurston Laboratory, Imperial College London[230])
ptCMV-EGFP-TRIM37	NP_001005207.1	130	*Homo sapiens*	PCR from mixed human cDNA, ptCMV-EGFP plasmid derived from pEGFP-N1 plasmid (Clontech) (T. Thurston Laboratory, Imperial College London[230])
ptCMV-EGFP-TRIM38	NP_006346.1	53	*Homo sapiens*	PCR from mixed human cDNA, ptCMV-EGFP plasmid derived from pEGFP-N1 plasmid (Clontech) (T. Thurston Laboratory, Imperial College London[230])
ptCMV-EGFP-TRIM39	NP_067076.2	60	*Homo sapiens*	PCR from mixed human cDNA, ptCMV-EGFP plasmid derived from pEGFP-N1 plasmid (Clontech) (T. Thurston Laboratory, Imperial College London[230])
ptCMV-EGFP-TRIM40	NP_001273562.1	29	*Homo sapiens*	PCR from mixed human cDNA, ptCMV-EGFP plasmid derived from pEGFP-N1 plasmid (Clontech) (T. Thurston Laboratory, Imperial College London[230])
ptCMV-EGFP-TRIM41	NP_291027.3	72	*Homo sapiens*	PCR from mixed human cDNA, ptCMV-EGFP plasmid derived from pEGFP-N1 plasmid (Clontech) (T. Thurston Laboratory, Imperial College London[230])
ptCMV-EGFP-TRIM42	NP_689829.3	80	*Homo sapiens*	PCR from mixed human cDNA, ptCMV-EGFP plasmid derived from pEGFP-N1 plasmid (Clontech) (T. Thurston Laboratory, Imperial College London[230])
ptCMV-EGFP-TRIM43	NP_620155.1	52	*Homo sapiens*	PCR from mixed human cDNA, ptCMV-EGFP plasmid derived from pEGFP-N1 plasmid (Clontech) (T. Thurston Laboratory, Imperial College London[230])
ptCMV-EGFP-TRIM44	NP_060053.2	38	*Homo sapiens*	PCR from mixed human cDNA, ptCMV-EGFP plasmid derived from pEGFP-N1 plasmid (Clontech) (T. Thurston Laboratory, Imperial College London[230])
ptCMV-EGFP-TRIM45	NP_079464.2	62	*Homo sapiens*	PCR from mixed human cDNA, ptCMV-EGFP plasmid derived from pEGFP-N1 plasmid (Clontech) (T. Thurston Laboratory, Imperial College London[230])

ptCMV-EGFP-TRIM46	NP_079334.3	83	*Homo sapiens*	Subcloned from M6P-EGFP vector generated by PCR of mixed human cDNA, ptCMV-EGFP plasmid derived from pEGFP-N1 plasmid (Clontech) (T. Thurston Laboratory, Imperial College London[230])
ptCMV-EGFP-TRIM47	NP_258411.2	70	*Homo sapiens*	PCR from mixed human cDNA, ptCMV-EGFP plasmid derived from pEGFP-N1 plasmid (Clontech) (T. Thurston Laboratory, Imperial College London[230])
ptCMV-EGFP-TRIM48	NP_077019.2	24	*Homo sapiens*	PCR from mixed human cDNA, ptCMV-EGFP plasmid derived from pEGFP-N1 plasmid (Clontech) (T. Thurston Laboratory, Imperial College London[230])
ptCMV-EGFP-TRIM49	NP_065091.1	53	*Homo sapiens*	PCR from mixed human cDNA, ptCMV-EGFP plasmid derived from pEGFP-N1 plasmid (Clontech) (T. Thurston Laboratory, Imperial College London[230])
ptCMV-EGFP-TRIM50	NP_001268380.1	55	*Homo sapiens*	Subcloned from plasmid gifted from Michaela Gack[27], ptCMV-EGFP plasmid derived from pEGFP-N1 plasmid (Clontech) (T. Thurston Laboratory, Imperial College London[230])
ptCMV-EGFP-TRIM51	NP_116070.2	53	*Homo sapiens*	PCR from mixed human cDNA, ptCMV-EGFP plasmid derived from pEGFP-N1 plasmid (Clontech) (T. Thurston Laboratory, Imperial College London[230])
ptCMV-EGFP-TRIM52	NP_116154.1	33	*Homo sapiens*	PCR from mixed human cDNA, ptCMV-EGFP plasmid derived from pEGFP-N1 plasmid (Clontech) (T. Thurston Laboratory, Imperial College London[230])
ptCMV-EGFP-TRIM54	NP_115935.3	40	*Homo sapiens*	PCR from plasmid synthesised by Open Biosystems, ptCMV-EGFP plasmid derived from pEGFP-N1 plasmid (Clontech) (T. Thurston Laboratory, Imperial College London[230])
ptCMV-EGFP-TRIM55	NP_908973.1	60	*Homo sapiens*	PCR from plasmid synthesised by Open Biosystems, ptCMV-EGFP plasmid derived from pEGFP-N1 plasmid (Clontech) (T. Thurston Laboratory, Imperial College London[230])
ptCMV-EGFP-TRIM56	NP_112223.1	81	*Homo sapiens*	PCR from mixed human cDNA, ptCMV-EGFP plasmid derived from pEGFP-N1 plasmid (Clontech) (T. Thurston Laboratory, Imperial College London[230])

ptCMV-EGFP-TRIM58	NP_056246.3	55	*Homo sapiens*	PCR from mixed human cDNA, ptCMV-EGFP plasmid derived from pEGFP-N1 plasmid (Clontech) (T. Thurston Laboratory, Imperial College London[230])
ptCMV-EGFP-TRIM59	NP_775107.1	47	*Homo sapiens*	PCR from mixed human cDNA, ptCMV-EGFP plasmid derived from pEGFP-N1 plasmid (Clontech) (T. Thurston Laboratory, Imperial College London[230])
ptCMV-EGFP-TRIM60	NP_001244954.1	55	*Homo sapiens*	Subcloned from M6P-EGFP vector generated by subcloning from plasmid synthesised by Open Biosystems, ptCMV-EGFP plasmid derived from pEGFP-N1 plasmid (Clontech) (T. Thurston Laboratory, Imperial College London[230])
ptCMV-EGFP-TRIM61	NP_001401833.1	24	*Homo sapiens*	PCR from plasmid synthesised by Open Biosystems, ptCMV-EGFP plasmid derived from pEGFP-N1 plasmid (Clontech) (T. Thurston Laboratory, Imperial College London[230])
ptCMV-EGFP-TRIM62	NP_060677.2	54	*Homo sapiens*	PCR from plasmid synthesised by Open Biosystems, ptCMV-EGFP plasmid derived from pEGFP-N1 plasmid (Clontech) (T. Thurston Laboratory, Imperial College London[230])
ptCMV-EGFP-TRIM63	NP_115977.2	40	*Homo sapiens*	PCR from mixed human cDNA, ptCMV-EGFP plasmid derived from pEGFP-N1 plasmid (Clontech) (T. Thurston Laboratory, Imperial College London[230])
ptCMV-EGFP-TRIM64	NP_001129958.1	52	*Homo sapiens*	PCR from mixed human cDNA, ptCMV-EGFP plasmid derived from pEGFP-N1 plasmid (Clontech) (T. Thurston Laboratory, Imperial College London[230])
ptCMV-EGFP-TRIM65	NP_775818.2	57	*Homo sapiens*	Subcloned from GenScript synthesised pcDNA-GFP vector, ptCMV-EGFP plasmid derived from pEGFP-N1 plasmid (Clontech) (T. Thurston Laboratory, Imperial College London[230])
ptCMV-EGFP-TRIM66	NP_001374951.1	135	*Homo sapiens*	PCR from mixed human cDNA, ptCMV-EGFP plasmid derived from pEGFP-N1 plasmid (Clontech) (T. Thurston Laboratory, Imperial College London[230])
ptCMV-EGFP-TRIM67	NP_001004342.3	84	*Homo sapiens*	Subcloned from GenScript synthesised pcDNA-GFP vector, ptCMV-EGFP plasmid derived from pEGFP-N1 plasmid (Clontech) (T. Thurston

				Laboratory, Imperial College London[230])
ptCMV-EGFP-TRIM68	NP_060543.5	56	*Homo sapiens*	PCR from mixed human cDNA, ptCMV-EGFP plasmid derived from pEGFP-N1 plasmid (Clontech) (T. Thurston Laboratory, Imperial College London[230])
ptCMV-EGFP-TRIM69	NP_892030.3	57	*Homo sapiens*	PCR from mixed human cDNA, ptCMV-EGFP plasmid derived from pEGFP-N1 plasmid (Clontech) (T. Thurston Laboratory, Imperial College London[230])
ptCMV-EGFP-TRIM70	NP_001340153.1	40	*Homo sapiens*	PCR from mixed human cDNA, ptCMV-EGFP plasmid derived from pEGFP-N1 plasmid (Clontech) (T. Thurston Laboratory, Imperial College London[230])
ptCMV-EGFP-TRIM71	NP_001034200.1	93	*Homo sapiens*	PCR from mixed human cDNA, ptCMV-EGFP plasmid derived from pEGFP-N1 plasmid (Clontech) (T. Thurston Laboratory, Imperial College London[230])
ptCMV-EGFP-TRIM72	NP_001008275.2	53	*Homo sapiens*	Subcloned from M6P-EGFP plasmid synthesised by Adolfo García-Sastre, , ptCMV-EGFP plasmid derived from pEGFP-N1 plasmid (Clontech) (T. Thurston Laboratory, Imperial College London[230])
ptCMV-EGFP-TRIM73	NP_944606.2	29	*Homo sapiens*	PCR from mixed human cDNA, ptCMV-EGFP plasmid derived from pEGFP-N1 plasmid (Clontech) (T. Thurston Laboratory, Imperial College London[230])
ptCMV-EGFP-TRIM74	NP_001304744.1	29	*Homo sapiens*	PCR from mixed human cDNA, ptCMV-EGFP plasmid derived from pEGFP-N1 plasmid (Clontech) (T. Thurston Laboratory, Imperial College London[230])
ptCMV-EGFP-TRIM75	NP_001382999.1	54	*Homo sapiens*	Subcloned from M6P-EGFP vector generated by PCR of mixed human cDNA, ptCMV-EGFP plasmid derived from pEGFP-N1 plasmid (Clontech) (T. Thurston Laboratory, Imperial College London[230])
ptCMV-EGFP-TRIM77	NP_001139634.1	52	*Homo sapiens*	Subcloned from M6P-EGFP vector generated by PCR of mixed human cDNA, ptCMV-EGFP plasmid derived from pEGFP-N1 plasmid (Clontech) (T. Thurston Laboratory, Imperial College London[230])
ptCMV-EGFP-TRIML1	NP_848651.2	53	*Homo sapiens*	Subcloned from M6P-EGFP vector generated by PCR of mixed human cDNA, ptCMV-EGFP plasmid derived from pEGFP-N1 plasmid (Clontech) (T.

				Thurston Laboratory, Imperial College London[230])
ptCMV-EGFP-TRIML2	NP_001290348.1	44	*Homo sapiens*	PCR from mixed human cDNA, ptCMV-EGFP plasmid derived from pEGFP-N1 plasmid (Clontech) (T. Thurston Laboratory, Imperial College London[230])

Supplementary References

1 Reymond, A. *et al.* The tripartite motif family identifies cell compartments. *The EMBO Journal* **20**, 2140-2151, (2001).

2 Stormo, A. E. D. *et al.* The E3 ligase TRIM1 ubiquitinates LRRK2 and controls its localization, degradation, and toxicity. *bioRxiv*, 2020.2010.2021.336578, (2020).

3 Buchner, G. *et al.* MID2, a Homologue of the Opitz Syndrome Gene MID1: Similarities in Subcellular Localization and Differences in Expression During Development. *Human Molecular Genetics* **8**, 1397-1407, (1999).

4 Short, K. M. & Cox, T. C. Subclassification of the RBCC/TRIM Superfamily Reveals a Novel Motif Necessary for Microtubule Binding. *Journal of Biological Chemistry* **281**, 8970-8980, (2006).

5 Ylikallio, E. *et al.* Deficiency of the E3 ubiquitin ligase TRIM2 in early-onset axonal neuropathy. *Human Molecular Genetics* **22**, 2975-2983, (2013).

6 Zhuang, T. *et al.* TRIM3 facilitates estrogen signaling and modulates breast cancer cell progression. *Cell Communication and Signaling* **20**, 45, (2022).

7 Tomar, D. *et al.* TRIM4; a novel mitochondrial interacting RING E3 ligase, sensitizes the cells to hydrogen peroxide (H2O2) induced cell death. *Free Radical Biology and Medicine* **89**, 1036-1048, (2015).

8 Fletcher, A. J. *et al.* Trivalent RING Assembly on Retroviral Capsids Activates TRIM5 Ubiquitination and Innate Immune Signaling. *Cell Host Microbe* **24**, 761-775.e766, (2018).

9 Diaz-Griffero, F. *et al.* Rapid turnover and polyubiquitylation of the retroviral restriction factor TRIM5. *Virology* **349**, 300-315, (2006).

10 Rajsbaum, R. *et al.* Unanchored K48-Linked Polyubiquitin Synthesized by the E3-Ubiquitin Ligase TRIM6 Stimulates the Interferon-IKKε Kinase-Mediated Antiviral Response. *Immunity* **40**, 880-895, (2014).

11 Montori-Grau, M. *et al.* GNIP1 E3 ubiquitin ligase is a novel player in regulating glycogen metabolism in skeletal muscle. *Metabolism* **83**, 177-187, (2018).

12 Jin, J. *et al.* E3 ubiquitin ligase TRIM7 negatively regulates NF-kappa B signaling pathway by degrading p65 in lung cancer. *Cellular Signalling* **69**, 109543, (2020).

13 Tian, Z. *et al.* TRIM8 inhibits breast cancer proliferation by regulating estrogen signaling. *Am J Cancer Res* **10**, 3440-3457, (2020).

14 Napolitano, Luisa M., Jaffray, Ellis G., Hay, Ronald T. & Meroni, G. Functional interactions between ubiquitin E2 enzymes and TRIM proteins. *Biochemical Journal* **434**, 309-319, (2011).

15 Guo, M. *et al.* TRIM10 binds to IFN-α/β receptor 1 to negatively regulate type I IFN signal transduction. *European Journal of Immunology* **51**, 1762-1773, (2021).

16 Chang, T.-H., Yoshimi, R. & Ozato, K. Tripartite Motif (TRIM) 12c, a Mouse Homolog of TRIM5, Is a Ubiquitin Ligase That Stimulates Type I IFN and NF-κB Pathways along with TNFR-Associated Factor 6. *The Journal of Immunology* **195**, 5367-5379, (2015).

17 Lascano, J., Uchil, P. D., Mothes, W. & Luban, J. TRIM5 Retroviral Restriction Activity Correlates with the Ability To Induce Innate Immune Signaling. *J Virol* **90**, 308-316, (2016).

18 Lerner, M. *et al.* The RBCC gene RFP2 (Leu5) encodes a novel transmembrane E3 ubiquitin ligase involved in ERAD. *Mol Biol Cell* **18**, 1670-1682, (2007).

19 Roy, M. *et al.* TNF-α-induced E3 ligase, TRIM15 inhibits TNF-α-regulated NF-κB pathway by promoting turnover of K63 linked ubiquitination of TAK1. *Cellular Signalling* **91**, 110210, (2022).

20 Lee, O. H. *et al.* Role of the focal adhesion protein TRIM15 in colon cancer development. *Biochim Biophys Acta* **1853**, 409-421, (2015).

21 Lassot, I. *et al.* Trim17, a novel E3 ubiquitin-ligase, initiates neuronal apoptosis. *Cell Death & Differentiation* **17**, 1928-1941, (2010).

22 Zanchetta, M. E., Napolitano, L. M. R., Maddalo, D. & Meroni, G. The E3 ubiquitin ligase MID1/TRIM18 promotes atypical ubiquitination of the BRCA2-associated factor

35, BRAF35. *Biochimica et Biophysica Acta (BBA) - Molecular Cell Research* **1864**, 1844-1854, (2017).

23 Sahin, U., Lallemand-Breitenbach, V. & de Thé, H. PML nuclear bodies: regulation, function and therapeutic perspectives. *The Journal of Pathology* **234**, 289-291, (2014).

24 Mallery, D. L. *et al.* Antibodies mediate intracellular immunity through tripartite motif-containing 21 (TRIM21). *Proceedings of the National Academy of Sciences* **107**, 19985-19990, (2010).

25 Kajaste-Rudnitski, A. *et al.* TRIM22 Inhibits HIV-1 Transcription Independently of Its E3 Ubiquitin Ligase Activity, Tat, and NF-kB-Responsive Long Terminal Repeat Elements. *Journal of Virology* **85**, 5183-5196, (2011).

26 Duan, Z., Gao, B., Xu, W. & Xiong, S. Identification of TRIM22 as a RING finger E3 ubiquitin ligase. *Biochemical and Biophysical Research Communications* **374**, 502-506, (2008).

27 Sparrer, K. M. J. *et al.* TRIM23 mediates virus-induced autophagy via activation of TBK1. *Nature Microbiology* **2**, 1543-1557, (2017).

28 Yu, T. *et al.* Modulation of M2 macrophage polarization by the crosstalk between Stat6 and Trim24. *Nature Communications* **10**, 4353, (2019).

29 Le Douarin, B. *et al.* The N-terminal part of TIF1, a putative mediator of the ligand-dependent activation function (AF-2) of nuclear receptors, is fused to B-raf in the oncogenic protein T18. *The EMBO Journal* **14**, 2020-2033, (1995).

30 Sanchez, J. G. *et al.* TRIM25 Binds RNA to Modulate Cellular Anti-viral Defense. *Journal of Molecular Biology* **430**, 5280-5293, (2018).

31 Li, X. *et al.* Deubiquitinase USP39 and E3 ligase TRIM26 balance the level of ZEB1 ubiquitination and thereby determine the progression of hepatocellular carcinoma. *Cell Death & Differentiation* **28**, 2315-2332, (2021).

32 Wang, P., Zhao, W., Zhao, K., Zhang, L. & Gao, C. TRIM26 Negatively Regulates Interferon-β Production and Antiviral Response through Polyubiquitination and Degradation of Nuclear IRF3. *PLOS Pathogens* **11**, e1004726, (2015).

33 Zhao, J. *et al.* TRIM26 positively regulates the inflammatory immune response through K11-linked ubiquitination of TAB1. *Cell Death & Differentiation* **28**, 3077-3091, (2021).

34 Zurek, B. *et al.* TRIM27 Negatively Regulates NOD2 by Ubiquitination and Proteasomal Degradation. *PLOS ONE* **7**, e41255, (2012).

35 Kato, T. *et al.* TRIM27/MRTF-B-Dependent Integrin β1 Expression Defines Leading Cells in Cancer Cell Collectives. *Cell Reports* **7**, 1156-1167, (2014).

36 Zhang, R.-Y. *et al.* UBE2S interacting with TRIM28 in the nucleus accelerates cell cycle by ubiquitination of p27 to promote hepatocellular carcinoma development. *Signal Transduction and Targeted Therapy* **6**, 64, (2021).

37 Qin, Y. *et al.* TRIM28 SUMOylates and stabilizes NLRP3 to facilitate inflammasome activation. *Nature Communications* **12**, 4794, (2021).

38 Choi, U. Y., Choi, W. Y., Hur, J. Y. & Kim, Y.-J. Polyubiquitin chain-dependent protein degradation in TRIM30 cytoplasmic bodies. *Experimental & Molecular Medicine* **47**, e159-e159, (2015).

39 Guo, P. *et al.* TRIM31 is upregulated in hepatocellular carcinoma and promotes disease progression by inducing ubiquitination of TSC1–TSC2 complex. *Oncogene* **37**, 478-488, (2018).

40 Song, H. *et al.* The E3 ubiquitin ligase TRIM31 attenuates NLRP3 inflammasome activation by promoting proteasomal degradation of NLRP3. *Nature Communications* **7**, 13727, (2016).

41 Sugiura, T. & Miyamoto, K. Characterization of TRIM31, upregulated in gastric adenocarcinoma, as a novel RBCC protein. *Journal of Cellular Biochemistry* **105**, 1081-1091, (2008).

42 Horn, E. J. *et al.* RING protein Trim32 associated with skin carcinogenesis has anti-apoptotic and E3-ubiquitin ligase properties. *Carcinogenesis* **25**, 157-167, (2004).

43 Lazzari, E. *et al.* Analysis of the Zn-Binding Domains of TRIM32, the E3 Ubiquitin Ligase Mutated in Limb Girdle Muscular Dystrophy 2H. *Cells* **8**, (2019).

44 Overå, K. S. *et al.* TRIM32, but not its muscular dystrophy-associated mutant, positively regulates and is targeted to autophagic degradation by p62/SQSTM1. *Journal of Cell Science* **132**, (2019).

45 Ryu, Y. S. *et al.* TRIM32 Protein Sensitizes Cells to Tumor Necrosis Factor-induced Apoptosis via Its RING Domain-dependent E3 Ligase Activity against X-linked Inhibitor of Apoptosis (XIAP). *Journal of Biological Chemistry* **286**, 25729-25738, (2011).

46 Gonzalez-Cano, L. *et al.* Regulatory feedback loop between TP73 and TRIM32. *Cell Death & Disease* **4**, e704-e704, (2013).

47 Tanaka, S. *et al.* Trim33 mediates the proinflammatory function of Th17 cells. *J Exp Med* **215**, 1853-1868, (2018).

48 Kulkarni, A. *et al.* Tripartite Motif-containing 33 (TRIM33) Protein Functions in the Poly(ADP-ribose) Polymerase (PARP)-dependent DNA Damage Response through Interaction with Amplified in Liver Cancer 1 (ALC1) Protein. *Journal of Biological Chemistry* **288**, 32357-32369, (2013).

49 Zou, D.-b., Mou, Z., Wu, W. & Liu, H. TRIM33 protects osteoblasts from oxidative stress-induced apoptosis in osteoporosis by inhibiting FOXO3a ubiquitylation and degradation. *Aging Cell* **20**, e13367, (2021).

50 Ohainle, M. *et al.* TRIM34 restricts HIV-1 and SIV capsids in a TRIM5α-dependent manner. *PLoS Pathog* **16**, e1008507, (2020).

51 Chen, Z. *et al.* Co-expression of PKM2 and TRIM35 predicts survival and recurrence in hepatocellular carcinoma. *Oncotarget* **6**, 2538-2548, (2015).

52 van Beuningen, Sam F. B. *et al.* TRIM46 Controls Neuronal Polarity and Axon Specification by Driving the Formation of Parallel Microtubule Arrays. *Neuron* **88**, 1208-1226, (2015).

53 Miyajima, N., Maruyama, S., Nonomura, K. & Hatakeyama, S. TRIM36 interacts with the kinetochore protein CENP-H and delays cell cycle progression. *Biochemical and Biophysical Research Communications* **381**, 383-387, (2009).

54 Chen, S. *et al.* TRIM37 Mediates Chemoresistance and Maintenance of Stemness in Pancreatic Cancer Cells via Ubiquitination of PTEN and Activation of the AKT–GSK-3β–β-Catenin Signaling Pathway. *Frontiers in Oncology* **10**, (2020).

55 Hämäläinen, R. *et al.* Wilms' tumor and novel TRIM37 mutations in an Australian patient with mulibrey nanism. *Clinical Genetics* **70**, 473-479, (2006).

56 Miao, C. *et al.* TRIM37 orchestrates renal cell carcinoma progression via histone H2A ubiquitination-dependent manner. *Journal of Experimental & Clinical Cancer Research* **40**, 195, (2021).

57 Wu, G. *et al.* An ATM/TRIM37/NEMO Axis Counteracts Genotoxicity by Activating Nuclear-to-Cytoplasmic NF-κB Signaling. *Cancer Research* **78**, 6399-6412, (2018).

58 Kallijärvi, J. *et al.* TRIM37 defective in mulibrey nanism is a novel RING finger ubiquitin E3 ligase. *Experimental Cell Research* **308**, 146-155, (2005).

59 Liu, X. *et al.* Enterovirus 71 induces degradation of TRIM38, a potential E3 ubiquitin ligase. *Virology Journal* **8**, 61, (2011).

60 Lee, S. S. *et al.* TRIM39 is a MOAP-1-binding protein that stabilizes MOAP-1 through inhibition of its poly-ubiquitination process. *Experimental Cell Research* **315**, 1313-1325, (2009).

61 Noguchi, K. *et al.* TRIM40 promotes neddylation of IKKγ and is downregulated in gastrointestinal cancers. *Carcinogenesis* **32**, 995-1004, (2011).

62 Lassot, I. *et al.* The E3 Ubiquitin Ligases TRIM17 and TRIM41 Modulate α-Synuclein Expression by Regulating ZSCAN21. *Cell Reports* **25**, 2484-2496.e2489, (2018).

63 Patil, G. *et al.* TRIM41-Mediated Ubiquitination of Nucleoprotein Limits Influenza A Virus Infection. *Journal of Virology* **92**, e00905-00918, (2018).

64 Yu, Z. *et al.* TRIM41 is required to innate antiviral response by polyubiquitinating BCL10 and recruiting NEMO. *Signal Transduction and Targeted Therapy* **6**, 90, (2021).

65 Full, F. *et al.* Centrosomal protein TRIM43 restricts herpesvirus infection by regulating nuclear lamina integrity. *Nature Microbiology* **4**, 164-176, (2019).

66 Sato, T., Takahashi, H., Hatakeyama, S., Iguchi, A. & Ariga, T. The TRIM-FLMN protein TRIM45 directly interacts with RACK1 and negatively regulates PKC-mediated signaling pathway. *Oncogene* **34**, 1280-1291, (2015).

67 Xia, Q., Zhan, G., Mao, M., Zhao, Y. & Li, X. TRIM45 causes neuronal damage by aggravating microglia-mediated neuroinflammation upon cerebral ischemia and reperfusion injury. *Experimental & Molecular Medicine* **54**, 180-193, (2022).

68 Harterink, M. *et al.* TRIM46 Organizes Microtubule Fasciculation in the Axon Initial Segment. *The Journal of Neuroscience* **39**, 4864-4873, (2019).

69 Ichinose, S., Ogawa, T., Jiang, X. & Hirokawa, N. The Spatiotemporal Construction of the Axon Initial Segment via KIF3/KAP3/TRIM46 Transport under MARK2 Signaling. *Cell Reports* **28**, 2413-2426.e2417, (2019).

70 Liang, Q. *et al.* TRIM47 is up-regulated in colorectal cancer, promoting ubiquitination and degradation of SMAD4. *Journal of Experimental & Clinical Cancer Research* **38**, 159, (2019).

71 Ma, X. *et al.* TRIM50 suppressed hepatocarcinoma progression through directly targeting SNAIL for ubiquitous degradation. *Cell Death & Disease* **9**, 608, (2018).

72 Qiu, Y. *et al.* TRIM50 acts as a novel Src suppressor and inhibits ovarian cancer progression. *Biochim Biophys Acta Mol Cell Res* **1866**, 1412-1420, (2019).

73 Fusco, C. *et al.* TRIM50 regulates Beclin 1 proautophagic activity. *Biochimica et Biophysica Acta (BBA) - Molecular Cell Research* **1865**, 908-919, (2018).

74 Fusco, C. *et al.* HDAC6 mediates the acetylation of TRIM50. *Cellular Signalling* **26**, 363-369, (2014).

75 Fusco, C. *et al.* The E3-Ubiquitin Ligase TRIM50 Interacts with HDAC6 and p62, and Promotes the Sequestration and Clearance of Ubiquitinated Proteins into the Aggresome. *PLOS ONE* **7**, e40440, (2012).

76 Micale, L. *et al.* Williams–Beuren syndrome TRIM50 encodes an E3 ubiquitin ligase. *European Journal of Human Genetics* **16**, 1038-1049, (2008).

77 Fan, W. *et al.* TRIM52: A nuclear TRIM protein that positively regulates the nuclear factor-kappa B signaling pathway. *Molecular Immunology* **82**, 114-122, (2017).

78 Pizon, V. *et al.* MURF2B, a Novel LC3-Binding Protein, Participates with MURF2A in the Switch between Autophagy and Ubiquitin Proteasome System during Differentiation of C2C12 Muscle Cells. *PLOS ONE* **8**, e76140, (2013).

79 Perera, S., Holt, M. R., Mankoo, B. S. & Gautel, M. Developmental regulation of MURF ubiquitin ligases and autophagy proteins nbr1, p62/SQSTM1 and LC3 during cardiac myofibril assembly and turnover. *Developmental Biology* **351**, 46-61, (2011).

80 Perera, S., Mankoo, B. & Gautel, M. Developmental regulation of MURF E3 ubiquitin ligases in skeletal muscle. *Journal of Muscle Research and Cell Motility* **33**, 107-122, (2012).

81 Wang, J. *et al.* TRIM56 Is a Virus- and Interferon-Inducible E3 Ubiquitin Ligase That Restricts Pestivirus Infection. *Journal of Virology* **85**, 3733-3745, (2011).

82 Xue, M. *et al.* Regulation of estrogen signaling and breast cancer proliferation by an ubiquitin ligase TRIM56. *Oncogenesis* **8**, 30, (2019).

83 Thom, Christopher S. *et al.* Trim58 Degrades Dynein and Regulates Terminal Erythropoiesis. *Developmental Cell* **30**, 688-700, (2014).

84 Kajiura, K. *et al.* Frequent silencing of the candidate tumor suppressor TRIM58 by promoter methylation in early-stage lung adenocarcinoma. *Oncotarget* **8**, 2890-2905, (2017).

85 Kondo, T., Watanabe, M. & Hatakeyama, S. TRIM59 interacts with ECSIT and negatively regulates NF-κB and IRF-3/7-mediated signal pathways. *Biochemical and Biophysical Research Communications* **422**, 501-507, (2012).

86 Huang, C.-J., Huang, C.-C. & Chang, C.-C. Association of the testis-specific TRIM/RBCC protein RNF33/TRIM60 with the cytoplasmic motor proteins KIF3A and KIF3B. *Molecular and Cellular Biochemistry* **360**, 121-131, (2012).

87 Schmidt, F. *et al.* The E3 ubiquitin ligase TRIM62 and inflammation-induced skeletal muscle atrophy. *Critical Care* **18**, 545, (2014).

88 Huang, F., Xiao, H., Sun, B.-L. & Yang, R.-G. Characterization of TRIM62 as a RING finger E3 ubiquitin ligase and its subcellular localization. *Biochemical and Biophysical Research Communications* **432**, 208-213, (2013).

89 Chen, S. N. *et al.* Human molecular genetic and functional studies identify TRIM63, encoding Muscle RING Finger Protein 1, as a novel gene for human hypertrophic cardiomyopathy. *Circ Res* **111**, 907-919, (2012).

90 Rodríguez, J. E. *et al.* The ubiquitin ligase MuRF1 regulates PPARα activity in the heart by enhancing nuclear export via monoubiquitination. *Molecular and Cellular Endocrinology* **413**, 36-48, (2015).

91 Khan, M. M. *et al.* Role of autophagy, SQSTM1, SH3GLB1, and TRIM63 in the turnover of nicotinic acetylcholine receptors. *Autophagy* **10**, 123-136, (2014).

92 Mattox, T. A. *et al.* MuRF1 activity is present in cardiac mitochondria and regulates reactive oxygen species production in vivo. *Journal of Bioenergetics and Biomembranes* **46**, 173-187, (2014).

93 Lang, X. *et al.* TRIM65-catalized ubiquitination is essential for MDA5-mediated antiviral innate immunity. *Journal of Experimental Medicine* **214**, 459-473, (2016).

94 Wei, W.-S. *et al.* TRIM65 supports bladder urothelial carcinoma cell aggressiveness by promoting ANXA2 ubiquitination and degradation. *Cancer Letters* **435**, 10-22, (2018).

95 Yang, Y.-F., Zhang, M.-F., Tian, Q.-H. & Zhang, C. Z. TRIM65 triggers β-catenin signaling via ubiquitylation of Axin1 to promote hepatocellular carcinoma. *Journal of Cell Science* **130**, 3108-3115, (2017).

96 Menon, S. *et al.* The TRIM9/TRIM67 neuronal interactome reveals novel activators of morphogenesis. *Molecular Biology of the Cell* **32**, 314-330, (2021).

97 Yaguchi, H. *et al.* TRIM67 Protein Negatively Regulates Ras Activity through Degradation of 80K-H and Induces Neuritogenesis. *Journal of Biological Chemistry* **287**, 12050-12059, (2012).

98 Wynne, C. *et al.* TRIM68 Negatively Regulates IFN-β Production by Degrading TRK Fused Gene, a Novel Driver of IFN-β Downstream of Anti-Viral Detection Systems. *PLOS ONE* **9**, e101503, (2014).

99 Han, R. *et al.* Trim69 regulates zebrafish brain development by ap-1 pathway. *Scientific Reports* **6**, 24034, (2016).

100 Han, Y., Li, R., Gao, J., Miao, S. & Wang, L. Characterisation of human RING finger protein TRIM69, a novel testis E3 ubiquitin ligase and its subcellular localisation. *Biochemical and Biophysical Research Communications* **429**, 6-11, (2012).

101 Wang, K. *et al.* Interferon-stimulated TRIM69 interrupts dengue virus replication by ubiquitinating viral nonstructural protein 3. *PLOS Pathogens* **14**, e1007287, (2018).

102 Chang, H.-M. *et al.* Trim71 cooperates with microRNAs to repress Cdkn1a expression and promote embryonic stem cell proliferation. *Nature Communications* **3**, 923, (2012).

103 Rybak, A. *et al.* The let-7 target gene mouse lin-41 is a stem cell specific E3 ubiquitin ligase for the miRNA pathway protein Ago2. *Nature Cell Biology* **11**, 1411-1420, (2009).

104 Cong, X. *et al.* TRIM72 promotes alveolar epithelial cell membrane repair and ameliorates lung fibrosis. *Respiratory Research* **21**, 132, (2020).

105 Kim, S. C. *et al.* TRIM72 is required for effective repair of alveolar epithelial cell wounding. *American Journal of Physiology-Lung Cellular and Molecular Physiology* **307**, L449-L459, (2014).

106 Jin, Z. *et al.* Efficient Gene Knockdown in Mouse Oocytes through Peptide Nanoparticle-Mediated SiRNA Transfection. *PLOS ONE* **11**, e0150462, (2016).

107 Balastik, M. *et al.* Deficiency in ubiquitin ligase TRIM2 causes accumulation of neurofilament light chain and neurodegeneration. *Proceedings of the National Academy of Sciences* **105**, 12016-12021, (2008).

108 Raheja, R., Liu, Y., Hukkelhoven, E., Yeh, N. & Koff, A. The ability of TRIM3 to induce growth arrest depends on RING-dependent E3 ligase activity. *Biochemical Journal* **458**, 537-545, (2014).

109 Zhu, J. *et al.* Targeting TRIM3 deletion-induced tumor-associated lymphangiogenesis prohibits lymphatic metastasis in esophageal squamous cell carcinoma. *Oncogene* **38**, 2736-2749, (2019).

110 Hung, A. Y., Sung, C. C., Brito, I. L. & Sheng, M. Degradation of Postsynaptic Scaffold GKAP and Regulation of Dendritic Spine Morphology by the TRIM3 Ubiquitin Ligase in Rat Hippocampal Neurons. *PLOS ONE* **5**, e9842, (2010).

111 Yan, J., Li, Q., Mao, A.-P., Hu, M.-M. & Shu, H.-B. TRIM4 modulates type I interferon induction and cellular antiviral response by targeting RIG-I for K63-linked ubiquitination. *Journal of Molecular Cell Biology* **6**, 154-163, (2014).

112 Liu, W. *et al.* The Expression of TRIM6 Activates the mTORC1 Pathway by Regulating the Ubiquitination of TSC1-TSC2 to Promote Renal Fibrosis. *Front Cell Dev Biol* **8**, 616747, (2020).

113 Zhu, L. *et al.* The E3 ubiquitin ligase TRIM7 suppressed hepatocellular carcinoma progression by directly targeting Src protein. *Cell Death Differ* **27**, 1819-1831, (2020).

114 Chakraborty, A., Diefenbacher, M. E., Mylona, A., Kassel, O. & Behrens, A. The E3 ubiquitin ligase Trim7 mediates c-Jun/AP-1 activation by Ras signalling. *Nat Commun* **6**, 6782, (2015).

115 Okumura, F., Matsunaga, Y., Katayama, Y., Nakayama, K. I. & Hatakeyama, S. TRIM8 modulates STAT3 activity through negative regulation of PIAS3. *Journal of Cell Science* **123**, 2238-2245, (2010).

116 Tanji, K. *et al.* TRIM9, a novel brain-specific E3 ubiquitin ligase, is repressed in the brain of Parkinson's disease and dementia with Lewy bodies. *Neurobiology of Disease* **38**, 210-218, (2010).

117 Yang, H. *et al.* Tripartite motif 10 regulates cardiac hypertrophy by targeting the PTEN/AKT pathway. *Journal of Cellular and Molecular Medicine* **24**, 6233-6241, (2020).

118 Huang, Q., Zhu, X. & Xu, M. Silencing of TRIM10 alleviates apoptosis in cellular model of Parkinson's disease. *Biochemical and Biophysical Research Communications* **518**, 451-458, (2019).

119 Zhu, G., Herlyn, M. & Yang, X. TRIM15 and CYLD regulate ERK activation via lysine-63-linked polyubiquitination. *Nature Cell Biology* **23**, 978-991, (2021).

120 Sun, Y. *et al.* TRIM15 promotes the invasion and metastasis of pancreatic cancer cells by mediating APOA1 ubiquitination and degradation. *Biochimica et Biophysica Acta (BBA) - Molecular Basis of Disease* **1867**, 166213, (2021).

121 Urano, T. *et al.* TRIM44 interacts with and stabilizes terf, a TRIM ubiquitin E3 ligase. *Biochemical and Biophysical Research Communications* **383**, 263-268, (2009).

122 Han, X., Du, H. & Massiah, M. A. Detection and Characterization of the In Vitro E3 Ligase Activity of the Human MID1 Protein. *Journal of Molecular Biology* **407**, 505-520, (2011).

123 Gu, H. & Roizman, B. The degradation of promyelocytic leukemia and Sp100 proteins by herpes simplex virus 1 is mediated by the ubiquitin-conjugating enzyme UbcH5a. *Proceedings of the National Academy of Sciences* **100**, 8963-8968, (2003).

124 Wada, K. & Kamitani, T. Autoantigen Ro52 is an E3 ubiquitin ligase. *Biochemical and Biophysical Research Communications* **339**, 415-421, (2006).

125 Kong, H. J. *et al.* Cutting Edge: Autoantigen Ro52 Is an Interferon Inducible E3 Ligase That Ubiquitinates IRF-8 and Enhances Cytokine Expression in Macrophages. *The Journal of Immunology* **179**, 26-30, (2007).

126 Ji, J. *et al.* TRIM22 activates NF-κB signaling in glioblastoma by accelerating the degradation of IκBα. *Cell Death & Differentiation* **28**, 367-381, (2021).

127 Vichi, A., Payne, D. M., Pacheco-Rodriguez, G., Moss, J. & Vaughan, M. E3 ubiquitin ligase activity of the trifunctional ARD1 (ADP-ribosylation factor domain protein 1). *Proceedings of the National Academy of Sciences* **102**, 1945-1950, (2005).

128 Watanabe, M. *et al.* The E3 ubiquitin ligase TRIM23 regulates adipocyte differentiation via stabilization of the adipogenic activator PPARγ. *eLife* **4**, e05615, (2015).

129 Han, Y. *et al.* TRIM23 overexpression is a poor prognostic factor and contributes to carcinogenesis in colorectal cancer. *Journal of Cellular and Molecular Medicine* **24**, 5491-5500, (2020).

130 Cao, X. *et al.* Opposing roles of E3 ligases TRIM23 and TRIM21 in regulation of ion channel ANO1 protein levels. *J Biol Chem* **296**, 100738, (2021).

131 Stevens, R. V., Esposito, D. & Rittinger, K. Characterisation of class VI TRIM RING domains: linking RING activity to C-terminal domain identity. *Life Science Alliance* **2**, e201900295, (2019).

132 Allton, K. *et al.* Trim24 targets endogenous p53 for degradation. *Proceedings of the National Academy of Sciences* **106**, 11612-11616, (2009).

133 Zhu, Q. *et al.* TRIM24 facilitates antiviral immunity through mediating K63-linked TRAF3 ubiquitination. *Journal of Experimental Medicine* **217**, (2020).

134 Gack, M. U. *et al.* TRIM25 RING-finger E3 ubiquitin ligase is essential for RIG-I-mediated antiviral activity. *Nature* **446**, 916-920, (2007).

135 Koliopoulos, M. G. *et al.* Molecular mechanism of influenza A NS1-mediated TRIM25 recognition and inhibition. *Nature Communications* **9**, 1820, (2018).

136 Koliopoulos, M. G., Esposito, D., Christodoulou, E., Taylor, I. A. & Rittinger, K. Functional role of TRIM E3 ligase oligomerization and regulation of catalytic activity. *EMBO J* **35**, 1204-1218, (2016).

137 Li, F. *et al.* OTUD5 cooperates with TRIM25 in transcriptional regulation and tumor progression via deubiquitination activity. *Nature Communications* **11**, 4184, (2020).

138 Williams Sarah, C. & Parsons Jason, L. NTH1 Is a New Target for Ubiquitylation-Dependent Regulation by TRIM26 Required for the Cellular Response to Oxidative Stress. *Molecular and Cellular Biology* **38**, e00616-00617, (2018).

139 Edmonds, M. J., Carter, R. J., Nickson, C. M., Williams, S. C. & Parsons, J. L. Ubiquitylation-dependent regulation of NEIL1 by Mule and TRIM26 is required for the cellular DNA damage response. *Nucleic Acids Research* **45**, 726-738, (2017).

140 Nakagawa, T. *et al.* Transforming Growth Factor β-Induced Proliferative Arrest Mediated by TRIM26-Dependent TAF7 Degradation and Its Antagonism by MYC. *Molecular and Cellular Biology* **38**, e00449-00417, (2018).

141 Ran, Y. *et al.* Autoubiquitination of TRIM26 links TBK1 to NEMO in RLR-mediated innate antiviral immune response. *Journal of Molecular Cell Biology* **8**, 31-43, (2016).

142 Miao, X. *et al.* TRIM27 promotes IL-6-induced proliferation and inflammation factor production by activating STAT3 signaling in HaCaT cells. *Am J Physiol Cell Physiol* **318**, C272-c281, (2020).

143 Xing, L. *et al.* TRIM27 Functions as a Novel Oncogene in Non-Triple-Negative Breast Cancer by Blocking Cellular Senescence through p21 Ubiquitination. *Molecular Therapy - Nucleic Acids* **22**, 910-923, (2020).

144 Li, Y., Meng, Q., Wang, L. & Cui, Y. TRIM27 protects against cardiac ischemia-reperfusion injury by suppression of apoptosis and inflammation via negatively regulating p53. *Biochemical and Biophysical Research Communications* **557**, 127-134, (2021).

145 Zaman, M. M.-U., Shinagawa, T. & Ishii, S. Trim27-deficient mice are susceptible to streptozotocin-induced diabetes. *FEBS Open Bio* **4**, 60-64, (2014).

146 Zheng, Q. *et al.* Siglec1 suppresses antiviral innate immune response by inducing TBK1 degradation via the ubiquitin ligase TRIM27. *Cell Research* **25**, 1121-1136, (2015).

147 Doyle, J. M., Gao, J., Wang, J., Yang, M. & Potts, P. R. MAGE-RING Protein Complexes Comprise a Family of E3 Ubiquitin Ligases. *Molecular Cell* **39**, 963-974, (2010).

148 Bian, H. *et al.* The E3 ubiquitin ligase MuRF2 attenuates LPS-induced macrophage activation by inhibiting production of inflammatory cytokines and migration. *FEBS Open Bio* **8**, 234-243, (2018).

149 Li, W., Li, F., Lei, W. & Tao, Z. TRIM30 modulates Interleukin-22-regulated papillary thyroid Cancer cell migration and invasion by targeting Sox17 for K48-linked Polyubiquitination. *Cell Communication and Signaling* **17**, 162, (2019).

150 Liu, B. *et al.* The ubiquitin E3 ligase TRIM31 promotes aggregation and activation of the signaling adaptor MAVS through Lys63-linked polyubiquitination. *Nature Immunology* **18**, 214-224, (2017).

151 Guo, Y. *et al.* Loss of TRIM31 promotes breast cancer progression through regulating K48- and K63-linked ubiquitination of p53. *Cell Death & Disease* **12**, 945, (2021).

152 Zhang, J. *et al.* The E3 ubiquitin ligase TRIM31 plays a critical role in hypertensive nephropathy by promoting proteasomal degradation of MAP3K7 in the TGF-β1 signaling pathway. *Cell Death & Differentiation* **29**, 556-567, (2022).

153 Kozakova, L. *et al.* The melanoma-associated antigen 1 (MAGEA1) protein stimulates the E3 ubiquitin-ligase activity of TRIM31 within a TRIM31-MAGEA1-NSE4 complex. *Cell Cycle* **14**, 920-930, (2015).

154 Yu, C., Chen, S., Guo, Y. & Sun, C. Oncogenic TRIM31 confers gemcitabine resistance in pancreatic cancer via activating the NF-κB signaling pathway. *Theranostics* **8**, 3224-3236, (2018).

155 Sugiura, T. The cellular level of TRIM31, an RBCC protein overexpressed in gastric cancer, is regulated by multiple mechanisms including the ubiquitin—proteasome system. *Cell Biology International* **35**, 657-661, (2011).

156 Kudryashova, E., Kudryashov, D., Kramerova, I. & Spencer, M. J. Trim32 is a Ubiquitin Ligase Mutated in Limb Girdle Muscular Dystrophy Type 2H that Binds to Skeletal Muscle Myosin and Ubiquitinates Actin. *Journal of Molecular Biology* **354**, 413-424, (2005).

157 Lazzari, E. *et al.* Analysis of the Zn-Binding Domains of TRIM32, the E3 Ubiquitin Ligase Mutated in Limb Girdle Muscular Dystrophy 2H. *Cells* **8**, 254, (2019).

158 Izumi, H. & Kaneko, Y. Trim32 Facilitates Degradation of MYCN on Spindle Poles and Induces Asymmetric Cell Division in Human Neuroblastoma Cells. *Cancer Research* **74**, 5620-5630, (2014).

159 Horn, E. J. *et al.* RING protein Trim32 associated with skin carcinogenesis has anti-apoptotic and E3-ubiquitin ligase properties. *Carcinogenesis* **25**, 157-167, (2004).

160 Nicklas, S. *et al.* A complex of the ubiquitin ligase TRIM32 and the deubiquitinase USP7 balances the level of c-Myc ubiquitination and thereby determines neural stem cell fate specification. *Cell Death Differ* **26**, 728-740, (2019).

161 Xue, J. *et al.* Tumour suppressor TRIM33 targets nuclear β-catenin degradation. *Nature Communications* **6**, 6156, (2015).

162 Ali, H. *et al.* Cellular TRIM33 restrains HIV-1 infection by targeting viral integrase for proteasomal degradation. *Nature Communications* **10**, 926, (2019).

163 Wang, X. *et al.* TRIM34 modulates influenza virus-activated programmed cell death by targeting Z-DNA-binding protein 1 for K63-linked polyubiquitination. *Journal of Biological Chemistry* **298**, 101611, (2022).

164 Wang, Y. *et al.* TRIM35 negatively regulates TLR7- and TLR9-mediated type I interferon production by targeting IRF7. *FEBS Letters* **589**, 1322-1330, (2015).

165 Sun, N. *et al.* TRIM35 mediates protection against influenza infection by activating TRAF3 and degrading viral PB2. *Protein & Cell* **11**, 894-914, (2020).

166 Tan, X. *et al.* Suppression of DLBCL Progression by the E3 Ligase Trim35 Is Mediated by CLOCK Degradation and NK Cell Infiltration. *Journal of Immunology Research* **2021**, 9995869, (2021).

167 Li, C. *et al.* Porcine TRIM35 positively regulate TRAF3-mediated IFN-β production and inhibit Japanese encephalitis virus replication. *Developmental & Comparative Immunology* **127**, 104290, (2022).

168 Yan, H., Ma, X., Mi, Z., He, Z. & Rong, P. Extracellular Polysaccharide from Rhizopus nigricans Inhibits Hepatocellular Carcinoma via miR-494-3p/TRIM36 Axis and Cyclin E Ubiquitination. *J Clin Transl Hepatol* **10**, 608-619, (2022).

169 Bhatnagar, S. *et al.* TRIM37 is a new histone H2A ubiquitin ligase and breast cancer oncoprotein. *Nature* **516**, 116-120, (2014).

170 Li, Y. *et al.* Tripartite motif-containing 37 (TRIM37) promotes the aggressiveness of non-small-cell lung cancer cells by activating the NF-κB pathway. *The Journal of Pathology* **246**, 366-378, (2018).

171 Zhao, W., Wang, L., Zhang, M., Yuan, C. & Gao, C. E3 Ubiquitin Ligase Tripartite Motif 38 Negatively Regulates TLR-Mediated Immune Responses by Proteasomal Degradation of TNF Receptor-Associated Factor 6 in Macrophages. *The Journal of Immunology* **188**, 2567-2574, (2012).

172 Liu, X. *et al.* Enterovirus 71 induces degradation of TRIM38, a potential E3 ubiquitin ligase. *Virol J* **8**, 61, (2011).

173 Wang, X. *et al.* TRIM38 triggers the ubiquitination and degradation of glucose transporter type 1 (GLUT1) to restrict tumor progression in bladder cancer. *Journal of Translational Medicine* **19**, 508, (2021).

174 Kim, K., Kim, J. H., Kim, I., Seong, S. & Kim, N. TRIM38 regulates NF-κB activation through TAB2 degradation in osteoclast and osteoblast differentiation. *Bone* **113**, 17-28, (2018).

175 Huang, N.-J. *et al.* The Trim39 ubiquitin ligase inhibits APC/CCdh1-mediated degradation of the Bax activator MOAP-1. *Journal of Cell Biology* **197**, 361-367, (2012).

176 Zhang, L. *et al.* TRIM39 regulates cell cycle progression and DNA damage responses via stabilizing p21. *Proceedings of the National Academy of Sciences* **109**, 20937-20942, (2012).

177 Hu, J. *et al.* TRIM39 deficiency inhibits tumor progression and autophagic flux in colorectal cancer via suppressing the activity of Rab7. *Cell Death & Disease* **12**, 391, (2021).

178 Suzuki, M. *et al.* TRIM39 negatively regulates the NFκB-mediated signaling pathway through stabilization of Cactin. *Cellular and Molecular Life Sciences* **73**, 1085-1101, (2016).

179 Zhao, C. *et al.* The E3 Ubiquitin Ligase TRIM40 Attenuates Antiviral Immune Responses by Targeting MDA5 and RIG-I. *Cell Reports* **21**, 1613-1623, (2017).

180 Shen, J. *et al.* TRIM40 inhibits IgA1-induced proliferation of glomerular mesangial cells by inactivating NLRP3 inflammasome through ubiquitination. *Molecular Immunology* **140**, 225-232, (2021).

181 Shen, Y. *et al.* Riok3 inhibits the antiviral immune response by facilitating TRIM40-mediated RIG-I and MDA5 degradation. *Cell Reports* **35**, 109272, (2021).

182 Zhang, X. *et al.* ANXA10 promotes melanoma metastasis by suppressing E3 ligase TRIM41-directed PKD1 degradation. *Cancer Letters* **519**, 237-249, (2021).

183 Zhang, Z. *et al.* SNP rs4971059 predisposes to breast carcinogenesis and chemoresistance via TRIM46-mediated HDAC1 degradation. *The EMBO Journal* **40**, e107974, (2021).

184 Zhang, J. *et al.* TRIM45 functions as a tumor suppressor in the brain via its E3 ligase activity by stabilizing p53 through K63-linked ubiquitination. *Cell Death & Disease* **8**, e2831-e2831, (2017).

185 Jiang, W. *et al.* Tripartite Motif-Containing 46 Promotes Viability and Inhibits Apoptosis of Osteosarcoma Cells by Activating NF-B Signaling Through Ubiquitination of PPAR. *Oncol Res* **28**, 409-421, (2020).

186 Tantai, J., Pan, X., Chen, Y., Shen, Y. & Ji, C. TRIM46 activates AKT/HK2 signaling by modifying PHLPP2 ubiquitylation to promote glycolysis and chemoresistance of lung cancer cells. *Cell Death & Disease* **13**, 285, (2022).

187 Zhang, J., Qiu, Q., Wang, H., Chen, C. & Luo, D. TRIM46 contributes to high glucose-induced ferroptosis and cell growth inhibition in human retinal capillary endothelial cells by facilitating GPX4 ubiquitination. *Experimental Cell Research* **407**, 112800, (2021).

188 Ji, Y.-X. *et al.* The deubiquitinating enzyme cylindromatosis mitigates nonalcoholic steatohepatitis. *Nature Medicine* **24**, 213-223, (2018).

189 Li, L. *et al.* TRIM47 accelerates aerobic glycolysis and tumor progression through regulating ubiquitination of FBP1 in pancreatic cancer. *Pharmacological Research* **166**, 105429, (2021).

190 Chen, J.-x. *et al.* TRIM47 promotes malignant progression of renal cell carcinoma by degrading P53 through ubiquitination. *Cancer Cell International* **21**, 129, (2021).

191 Azuma, K. *et al.* TRIM47 activates NF-kB signaling via PKCb/PKD3 stabilization and contributes to endocrine therapy resistance in breast cancer. *Proceedings of the National Academy of Sciences* **118**, e2100784118, (2021).

192 Hirata, Y. *et al.* TRIM48 Promotes ASK1 Activation and Cell Death through Ubiquitination-Dependent Degradation of the ASK1-Negative Regulator PRMT1. *Cell Reports* **21**, 2447-2457, (2017).

193 Guimarães, D. S. & Gomes, M. D. Expression, purification, and characterization of the TRIM49 protein. *Protein Expression and Purification* **143**, 57-61, (2018).

194 Li, R. *et al.* TRIM50 Suppresses Pancreatic Cancer Progression and Reverses the Epithelial-Mesenchymal Transition via Facilitating the Ubiquitous Degradation of Snail1. *Frontiers in oncology* **11**, 695740-695740, (2021).

195 Zhang, Y. *et al.* Tripartite Motif Containing 52 (TRIM52) Promotes Cell Proliferation in Hepatitis B Virus-Associated Hepatocellular Carcinoma. *Med Sci Monit* **23**, 5202-5210, (2017).

196 Zhang, Y. *et al.* TRIM52 up-regulation in hepatocellular carcinoma cells promotes proliferation, migration and invasion through the ubiquitination of PPM1A. *Journal of Experimental & Clinical Cancer Research* **37**, 116, (2018).

197 Fan, W. *et al.* TRIM52 inhibits Japanese Encephalitis Virus replication by degrading the viral NS2A. *Scientific Reports* **6**, 33698, (2016).

198 Sun, J. *et al.* TRIM52 positively mediates NF-κB to promote the growth of human benign prostatic hyperplasia cells through affecting TRAF2 ubiquitination. *Life Sciences* **259**, 118380, (2020).

199 Quintana, M. T. *et al.* Muscle ring finger-3 protects against diabetic cardiomyopathy induced by a high fat diet. *BMC Endocrine Disorders* **15**, 36, (2015).

200 Fielitz, J. *et al.* Myosin accumulation and striated muscle myopathy result from the loss of muscle RING finger 1 and 3. *The Journal of Clinical Investigation* **117**, 2486-2495, (2007).

201 Zhu, J. *et al.* Targeting TRIM54/Axin1/β-Catenin Axis Prohibits Proliferation and Metastasis in Hepatocellular Carcinoma. *Frontiers in Oncology* **11**, (2021).

202 Cao, H. *et al.* Tripartite motif-containing 54 promotes gastric cancer progression by upregulating K63-linked ubiquitination of filamin C. *Asia-Pacific Journal of Clinical Oncology* **n/a**, (2022).

203 He, J. *et al.* MuRF2 regulates PPARγ1 activity to protect against diabetic cardiomyopathy and enhance weight gain induced by a high fat diet. *Cardiovascular Diabetology* **14**, 97, (2015).

204 Guo, T. *et al.* TRIM55 suppresses malignant biological behavior of lung adenocarcinoma cells by increasing protein degradation of Snail1. *Cancer Biology & Therapy* **23**, 17-26, (2022).

205 Seo, G. J. *et al.* TRIM56-mediated monoubiquitination of cGAS for cytosolic DNA sensing. *Nature Communications* **9**, 613, (2018).

206 Fiskin, E. *et al.* Structural basis for the recognition and degradation of host TRIM proteins by Salmonella effector SopA. *Nature Communications* **8**, 14004, (2017).

207 Eyking, A., Ferber, F., Köhler, S., Reis, H. & Cario, E. TRIM58 Restrains Intestinal Mucosal Inflammation by Negatively Regulating TLR2 in Myeloid Cells. *The Journal of Immunology* **203**, 1636-1649, (2019).

208 Wang, J. *et al.* TRIM58 inactivates p53/p21 to promote chemoresistance via ubiquitination of DDX3 in breast cancer. *The International Journal of Biochemistry & Cell Biology* **143**, 106140, (2022).

209 Yuan, P. *et al.* TRIM58 Interacts with Pyruvate Kinase M2 to Inhibit Tumorigenicity in Human Osteosarcoma Cells. *BioMed Research International* **2020**, 8450606, (2020).

210 Liu, X., Long, Z., Cai, H., Yu, S. & Wu, J. TRIM58 suppresses the tumor growth in gastric cancer by inactivation of β-catenin signaling via ubiquitination. *Cancer Biology & Therapy* **21**, 203-212, (2020).

211 Han, T. *et al.* TRIM59 regulates autophagy through modulating both the transcription and the ubiquitination of BECN1. *Autophagy* **14**, 2035-2048, (2018).

212 Liu, Y. *et al.* TRIM59 overexpression correlates with poor prognosis and contributes to breast cancer progression through AKT signaling pathway. *Molecular Carcinogenesis* **57**, 1792-1802, (2018).

213 Tan, P. *et al.* TRIM59 promotes breast cancer motility by suppressing p62-selective autophagic degradation of PDCD10. *PLOS Biology* **16**, e3000051, (2018).

214 Zhou, Z. *et al.* TRIM59 Is Up-regulated in Gastric Tumors, Promoting Ubiquitination and Degradation of p53. *Gastroenterology* **147**, 1043-1054, (2014).

215 Cao, Z. *et al.* Ubiquitin Ligase TRIM62 Regulates CARD9-Mediated Anti-fungal Immunity and Intestinal Inflammation. *Immunity* **43**, 715-726, (2015).

216 Liu, X. & Lei, Q. TRIM62 knockout protects against cerebral ischemic injury in mice by suppressing NLRP3-regulated neuroinflammation. *Biochemical and Biophysical Research Communications* **529**, 140-147, (2020).

217 Zhu, C. *et al.* TRIM64 promotes ox-LDL-induced foam cell formation, pyroptosis, and inflammation in THP-1-derived macrophages by activating a feedback loop with NF-κB via IκBα ubiquitination. *Cell Biology and Toxicology*, (2022).

218 Li, S. *et al.* TRIM65 regulates microRNA activity by ubiquitination of TNRC6. *Proc Natl Acad Sci U S A* **111**, 6970-6975, (2014).

219 Chen, D. *et al.* Ubiquitin ligase TRIM65 promotes colorectal cancer metastasis by targeting ARHGAP35 for protein degradation. *Oncogene* **38**, 6429-6444, (2019).

220 Li, Y. *et al.* TRIM65 negatively regulates p53 through ubiquitination. *Biochemical and Biophysical Research Communications* **473**, 278-282, (2016).

221 Boyer, N. P., McCormick, L. E., Menon, S., Urbina, F. L. & Gupton, S. L. A pair of E3 ubiquitin ligases compete to regulate filopodial dynamics and axon guidance. *Journal of Cell Biology* **219**, (2019).

222 Miyajima, N. *et al.* TRIM68 Regulates Ligand-Dependent Transcription of Androgen Receptor in Prostate Cancer Cells. *Cancer Research* **68**, 3486-3494, (2008).

223 Bagga, T., Tulsian, N. K., Mok, Y. K., Kini, R. M. & Sivaraman, J. Mapping of molecular interactions between human E3 ligase TRIM69 and Dengue virus NS3 protease using hydrogen-deuterium exchange mass spectrometry. *Cell Mol Life Sci* **79**, 233, (2022).

224 Rong, X. *et al.* TRIM69 inhibits cataractogenesis by negatively regulating p53. *Redox Biology* **22**, 101157, (2019).

225 Lee, S. H. *et al.* The ubiquitin ligase human TRIM71 regulates let-7 microRNA biogenesis via modulation of Lin28B protein. *Biochimica et Biophysica Acta (BBA) - Gene Regulatory Mechanisms* **1839**, 374-386, (2014).

226 Nguyen, D. T. T. *et al.* The ubiquitin ligase LIN41/TRIM71 targets p53 to antagonize cell death and differentiation pathways during stem cell differentiation. *Cell Death & Differentiation* **24**, 1063-1078, (2017).

227 Gupta, I., Dharadhar, S., Sixma, T. & Khan, S. Biochemical characterization of TRIM72 E3 ligase and its interaction with the insulin receptor substrate 1. *Biochemistry and Biophysics Reports* **21**, 100729, (2020).

228 Ma, X. *et al.* The E3 ubiquitin ligase MG53 inhibits hepatocellular carcinoma by targeting RAC1 signaling. *Oncogenesis* **11**, 40, (2022).

229 Esposito, D., Dudley-Fraser, J., Garza-Garcia, A. & Rittinger, K. Divergent self-association properties of paralogous proteins TRIM2 and TRIM3 regulate their E3 ligase activity. *Nature Communications* **13**, 7583, (2022).

230 Günster, R. A., Matthews, S. A., Holden, D. W. & Thurston, T. L. M. SseK1 and SseK3 Type III Secretion System Effectors Inhibit NF-κB Signaling and Necroptotic Cell Death in Salmonella-Infected Macrophages. *Infect Immun* **85**, e00010-00017, (2017).
